# Genomic Variation and Recent Population Histories of Spotted (*Strix occidentalis*) and Barred (*Strix varia*) Owls

**DOI:** 10.1093/gbe/evab066

**Published:** 2021-03-25

**Authors:** Naoko T Fujito, Zachary R Hanna, Michal Levy-Sakin, Rauri C K Bowie, Pui-Yan Kwok, John P Dumbacher, Jeffrey D Wall

**Affiliations:** 1 Institute for Human Genetics, University of California San Francisco, CA, USA; 2 Museum of Vertebrate Zoology, University of California Berkeley, CA, USA; 3 Cardiovascular Research Institute, University of California San Francisco, CA, USA; 4 Department of Integrative Biology, University of California Berkeley, CA, USA; 5 Department of Ornithology and Mammology, California Academy of Sciences, San Francisco, CA, USA

**Keywords:** population split, population structure, hybridization and conservation genetics

## Abstract

Spotted owls (SOs, *Strix occidentalis*) are a flagship species inhabiting old-growth forests in western North America. In recent decades, their populations have declined due to ongoing reductions in suitable habitat caused by logging, wildfires, and competition with the congeneric barred owl (BO, *Strix varia*). The northern spotted owl (*S. o. caurina*) has been listed as “threatened” under the Endangered Species Act since 1990. Here, we use an updated SO genome assembly along with 51 high-coverage whole-genome sequences to examine population structure, hybridization, and recent changes in population size in SO and BO. We found that potential hybrids identified from intermediate plumage morphology were a mixture of pure BO, F1 hybrids, and F1 × BO backcrosses. Also, although SO underwent a population bottleneck around the time of the Pleistocene–Holocene transition, their population sizes rebounded and show no evidence of any historical (i.e., 100–10,000 years ago) population decline. This suggests that the current decrease in SO abundance is due to events in the past century. Finally, we estimate that western and eastern BOs have been genetically separated for thousands of years, instead of the previously assumed recent (i.e., <150 years) divergence. Although this result is surprising, it is unclear where the ancestors of western BO lived after the separation. In particular, although BO may have colonized western North America much earlier than the first recorded observations, it is also possible that the estimated divergence time reflects unsampled BO population structure within central or eastern North America.

Significance

Spotted owls, a threatened species in western North America, have been the subject of conservation research for decades and have had a tremendous economic impact on the timber industry. Their primary threat is competition from the congeneric barred owl, which was originally native to eastern North America. We describe the largest ever genomic study of spotted owls, barred owls, and their hybrids, including an improved spotted owl genome assembly and 51 high-coverage whole-genome sequences. Our results shed new light on their population structure and hybridization. Most notably, we find that western and eastern barred owls have been isolated for thousands of years, in contrast to the conventional wisdom. This result may have important implications for future management strategies.

## Introduction

Spotted owls (SOs, *Strix occidentalis*) occupy forests in western North America. There are three recognized subspecies (Dawson et al. 1987; Fleischer et al. 2004; Barrowclough et al. 2005; Funk et al. 2008): the northern spotted owl (NSO, *S. o. caurina*), found from southern British Columbia southward to southern Marin County in California; the California spotted owl (CSO, *S. o. occidentalis*), found from approximately the Pit River in northern California southward through the Sierra Nevada ranges to Baja California, and northward along the coast ranges to San Francisco; and the Mexican spotted owl (MSO, *S. o. lucida*), found in Mexico and the sky island forests of the south-western US deserts. Populations of all three subspecies have been declining for decades, leading the US Fish and Wildlife Service (USFWS) to list the NSO and MSO as “threatened” under the Endangered Species Act in the early 1990s (Thomas et al. 1990). The CSO was also petitioned for listing recently, but the application was rejected by USFWS in November 2019.

The listing of NSO has led to changes in forest management practices across the Pacific Northwest, which have had an ongoing economic effect on the West Coast timber industry (Courtney et al. 2004). Although this act was initially motivated by concerns over habitat loss (Forsman et al. 1984; Anderson and Burnham 1992), it is now clear that competition with the congeneric, invasive barred owl (BO, *Strix varia*) poses an additional and perhaps greater threat (Diller et al. 2016; Dugger et al. 2016). Observational data suggest that BOs, previously inhabiting areas east of the Rocky Mountains and Great Plains, have expanded their range over the past 80–130 years (Dark and Gould, 1998; Livezey 2009a,b) to include western North America, where they are sympatric with and out-compete NSOs (Wiens et al. 2014). BOs continue to expand their range southward, currently overlapping with CSOs as far south as Kern County, near Bakersfield, California.

Previous genetic work estimated an average autosomal sequence divergence of 0.7% between SO and BO (Hanna et al. 2018). However, the two species have been shown to hybridize and backcross in the wild (Haig, Mullins, Forsman, Trail, et al. 2004; Kelly and Forsman 2004; Hanna et al. 2018), leading to the concern that the SO gene pool may eventually be diluted/swamped by BO DNA. So far, hybridization has been observed in areas where SOs greatly outnumber BOs (Kelly and Forsman 2004), whereas observed interspecies mating pairs mainly involved a female BO with a male SO (Hamer and Forsman 1994; Haig, Mullins, Forsman, Trail, et al. 2004; Kelly and Forsman 2004).

**Fig. 1.**
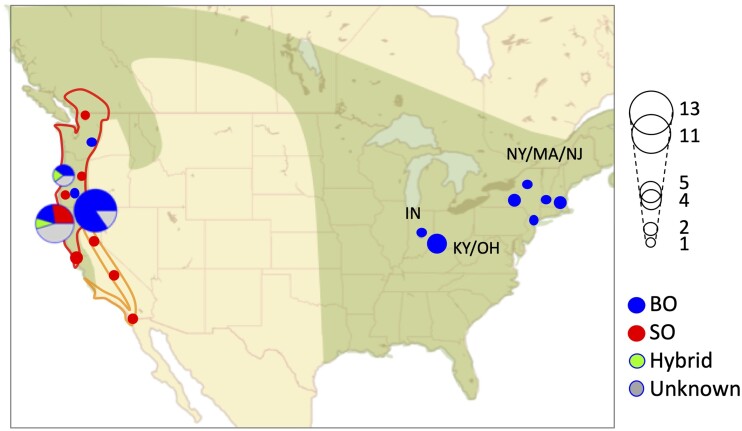
Geographic distribution of samples. Sampling locations of the 51 individuals in our study. Putative identities of samples categorized with sampling locations, morphology, and vocalization are shown: SOs (*Strix occidentalis*), BO (*S. varia*), and hybrids. Putative hybrids are shown as “unknown.” For locations with a high density of samples (e.g., Humboldt County and Siskiyou + Shasta County in California; Lane + Benton County in Oregon), the distribution of sampled individuals is visualized in pie charts. The size of circles and pie charts correspond to the number of samples. The range of BOs is shown in green. The ranges for NSO and CSO are shown with red and orange lines, respectively. Sampling locations of EBO in figure 2*C* were shown: Kentucky and Ohio (KY/OH), New York, Massachusetts, and New Jersey (NY/MA/NJ) and Indiana (IN).

We had previously speculated that the unusual plumage pattern seen in some western barred owls (WBO) was due to introgression with SO (see fig. 1, Hanna et al. 2018). However, analyses of low-coverage whole-genome sequence data from these birds suggested that the vast majority of these phenotypically unusual individuals were genetically purebred BO (Hanna et al. 2018). The question of how some WBO evolved a unique plumage pattern in such a short timeframe remains unclear. One possibility (which we explore in this study) is that WBOs may have (genetically) diverged from eastern barred owls (EBOs) more than 130 years ago, despite the lack of observational data of BO in western North America prior to the late 19th century.

In part to quantify any potential population structure within BO, we initiated a large-scale genomic study of SOs, BOs, and their hybrids. We generated an improved SO genome assembly (using data from 10x Genomics [10xG] and Bionano Genomics) and high-coverage whole-genome sequence data from 51 owls, including 8 NSO, 3 CSO, 12 EBO, 13 WBO, 2 known hybrids (identified in Hanna et al. 2018), and 13 potential hybrids. Our diverse sampling within species enabled us to quantify levels of population structure and divergence times within species, whereas the highly contiguous genome assembly enabled us to estimate past population sizes using information on how correlated patterns of diversity are as a function of physical distance along the chromosomes. Finally, our genetic characterization of the ancestry of potential hybrids allows us to directly test whether introgression is sex biased or not.

## Results

### New Assembly of *S. occidentalis*

We improved upon our previous SO genome, “StrOccCau_1.0” (Hanna, Henderson, Wall, et al. 2017), using 10xG linked-read data and Bionano Genomics optical maps. For the new assembly, we used the same female *S. occidentalis* sample named Sequoia (hereafter simply Sequoia) that was used to construct the previous assembly (Hanna, Henderson, Wall, et al. 2017). Our new data resulted in a more contiguous assembly, “StrOccCau_2.0” (supplementary table S1 and fig. S1, Supplementary Material online), with the N50 scaffold size increasing from 4.0 to 20.5 Mb.

We also used read depth information in males versus females to identify scaffolds lying on the Z or W chromosomes (supplementary figs. S2–S4 and tables S2–S4, Supplementary Material online). Out of the 97 scaffolds larger than 1 Mb, 15 are partial Z chromosome sequences and 82 are autosomal. The total lengths of scaffolds and contigs identified as autosomal, Z chromosome, and W chromosome are 1.09 Gb, 84.9 Mb, and 8.6 Mb, respectively. We restricted our analyses to the 82 large autosomal scaffolds.

### Description of the Data

We generated high-coverage (mean 31.70×, ± 6.51) whole-genome sequence data from 51 owl samples (including Sequoia) from various sampling locations (fig. 1 and supplementary table S5, Supplementary Material online). For convenience, we used simple informal identifiers for these samples; the corresponding museum IDs are shown in supplementary table S5, Supplementary Material online. These 51 samples consisted of 8 NSO, 3 CSO, 13 WBO, 12 EBO, 2 previously confirmed hybrids (cf. Hanna et al. 2018), and 13 putative hybrids. Nine individuals were classified as putative hybrids based on their unusual intermediate plumage pattern (Hanna et al. 2018), whereas four others had observational data on vocalization and behavior suggesting likely hybrid ancestry. We also verified the sex of each of the samples using the CHD1 locus, a commonly used avian sex marker (supplementary table S5, Supplementary Material online).

Standard pipelines were used to map reads to the new reference genome and call variants (see Materials and Methods). We identified 17,385,299 biallelic single nucleotide polymorphisms (SNPs) across the 82 large autosomal scaffolds, and 8,543,351 of these had high-confidence genotype calls (GQ ≥ 40) in all individuals.

### Population Structure

**Fig. 2.**
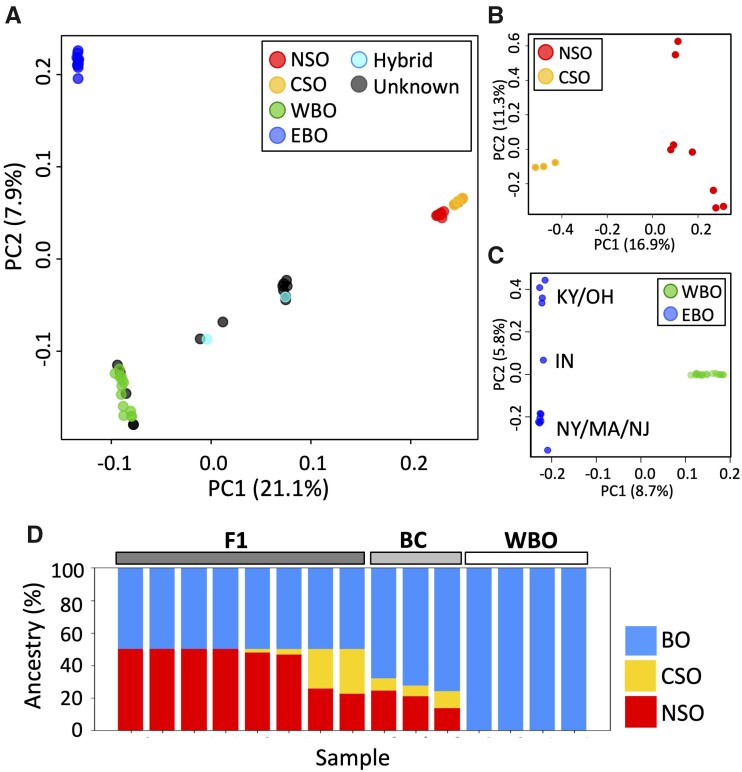
PCA for 51 samples. Colors indicate the primary identification of the samples based on morphology, vocalization, and sampling locations: NSO, CSO, WBOs, EBOs, hybrids, and putative hybrid (unknown) samples. (*A*) PCA for 11 SO samples. Clusters correspond to the two subspecies, NSO and CSO. (*B*) PCA for 29 BO samples. Colors indicate the genetic identification of the samples from figure 2*A*. Geographic locations of EBO were reflected in PC2. The sampling locations were shown as: Kentucky and Ohio (KY/OH), New York, Massachusetts, and New Jersey (NY/MA/NJ) and Indiana (IN). (*C*) Inferred ancestry of putative and genetically identified hybrids. Percentage of population-specific alleles is shown for each sample.

The range of morphological variation among hybrids and WBOs often makes it difficult to distinguish them from each other based solely on appearance. Subspecies of SO have been historically recognized based on body size, plumage coloration, and geographic range, but classification is not always clear (Haig, Mullins, Forsman et al. 2004; Barrowclough et al. 2005; Funk et al. 2008). We used principal component analysis (PCA) on all 51 samples to get a qualitative picture of population structure. SO, WBO, and EBO each cluster into well-defined groups, whereas hybrids are scattered between the SO and WBO clusters (fig. 2*A*). Among the 13 putative hybrids, 4 samples appear to be WBOs, and 9 are hybrids (supplementary table S5 and fig. S5, Supplementary Material online). This gives us a total of 17 WBO and 11 hybrid samples. Eight out of 11 hybrids were located in the middle on the *x* axis, and the other three hybrids are scattered in positions closer to WBO. Species-specific PCA plots show clear separation between CSO and NSO (fig. 2*B*), as well as some population structure within BO (fig. 2*C*). EBO substructure follows geography on PC2, with the one EBO that is “closest” to WBO from Indiana (fig. 2*C* and supplementary fig. S6, Supplementary Material online). The PCA patterns in figure 2*C* and supplementary figure S6, Supplementary Material online, are consistent with WBO being recently derived from EBO, with PC2 likely reflecting isolation by distance in EBO and PC1 reflecting the divergence between EBO and WBO. The nucleotide diversity calculated between each EBO sample and all the WBO samples is shown in supplementary figure S7, Supplementary Material online.

### Hybrid Characterization

We identified 2,501,269 apparent fixed differences between 11 SO and 25 morphologically identified BO. We then tabulated the genotypes at these sites for the putative and genetically identified hybrids to characterize their ancestry proportions. The four samples clustered together with WBO in figure 2*A* were homozygous for “BO alleles” at all these sites, confirming that they are pure BOs. The eight samples located in the middle of the *x* axis in figure 2*A* were heterozygous for (almost) all of these sites, which shows them to be first-generation hybrids (F1). The remaining three hybrids had 24–32% SO alleles at these fixed differences (fig. 2*D* and supplementary table S6*A*, Supplementary Material online), with no homozygous SO genotypes. We provisionally consider them to be F1 × BO backcrosses, since the exact SO ancestry percentages can fluctuate around the expectation of 25% due to recombination. However, the confirmation will require a genome assembly with whole-chromosome scaffolds to assess the size of the SO ancestry blocks in these individuals.

We used a similar approach to assess CSO versus NSO ancestry in hybrid individuals using 773 apparent fixed differences between NSO and CSO (that are not segregating in BO). Six out of eight F1s are NSO × BO offspring, consistent with their sampling locations (supplementary tables S5, S6*B*, Supplementary Material online and figs. 1 and 2*D*). The SO ancestry of the remaining hybrids though seems to contain substantial CSO and NSO ancestry components (supplementary table S6*B*, Supplementary Material online and fig. 2*D*).

We also inferred the ancestral components in samples using ADMIXTURE (Alexander et al. 2009) under a range of *K* values (*K* = 1–5) (Supplementary Materials and supplementary fig. S8, Supplementary Material online). *K* = 2, the most likely number of genetic components (supplementary fig. S8*A*, Supplementary Material online), separates SO from BO (supplementary fig. S8*B*, Supplementary Material online). The estimated SO proportions from the hybrids using ADMIXTURE (*K* = 2) are essentially identical to the results presented earlier in figure 2*D*.

### Diversity Analysis

After excluding first-degree relatives (Supplementary Materials and supplementary table S7, Supplementary Material online), we calculated genetic diversity for each population (supplementary table S8, Supplementary Material online). Consistent with an expected situation for threatened species, autosomal nucleotide diversities (π) of SOs were very small (1.41 × 10^−4^ for entire SO, 1.14 × 10^−4^ for NSO, and 1.48 × 10^−4^ for CSO), whereas the nucleotide diversities of BOs were more than 10 times higher (2.32 × 10^−3^ for entire BO, 2.15 × 10^−3^ for WBO, and 2.37 × 10^−3^ for EBO). WBO, which is hypothesized to have experienced a bottleneck during its recent invasion of the western US, showed a slightly smaller π value (2.15 × 10^−3^) than EBO (2.37 × 10^−3^). The nucleotide diversity between the two subspecies of SOs was 1.69 × 10^−4^, whereas the π between western and eastern populations of BOs was 2.37 × 10^−3^. *F*_ST_ between NSOs and CSOs was 0.253, whereas *F*_ST_ between EBOs and WBOs was 0.050, and both values are far smaller than the *F*_ST_ between the two species (0.765) (supplementary table S9, Supplementary Material online). Since both minor alleles and alleles with intermediate frequency can equally contribute to nucleotide diversity, π between populations reflects both the differentiation between the two populations and the population structure within each population. *F*_ST_ is commonly used for measuring differentiation between populations, though its estimator can be affected by the asymmetry in sample sizes of the populations (Bhatia et al. 2013). In this case, the numbers of individuals of WBO and EBO are roughly equal (13 and 12 samples, respectively), so *F*_ST_ should measure population differentiation reasonably accurately.

### Female Ancestry of Hybrids

It has been suggested that hybridization between SO and BO almost always involves male SOs pairing with female BOs (Hamer and Forsman 1994; Haig, Mullins, Forsman, Trail, et al. 2004; Kelly and Forsman 2004), although one hybrid carrying an SO haplotype of the mitochondrial control region was previously reported (Haig, Mullins, Forsman, Trail, et al. 2004). It was later found that both BO and SO have duplicated mitochondrial control regions (Hanna, Henderson, Sellas, et al. 2017), making it unclear whether the earlier genetic results were completely accurate. We traced the maternal ancestry of our hybrids through the noncoding region of mitochondrial DNA, to determine whether there were sex-biased hybridization patterns (see Supplementary Materials for details). Two out of 11 hybrids had SO mitochondrial DNA (indicating that their parents were a female SO and a male BO), whereas the remaining nine had BO mitochondrial DNA (supplementary fig. S9, Supplementary Material online). Although the sample size is low, our results suggest that there is some sex bias in SO × BO pairings toward pairings between male SO and female BO.

### Inference of Historical Population Sizes

**Fig. 3.**
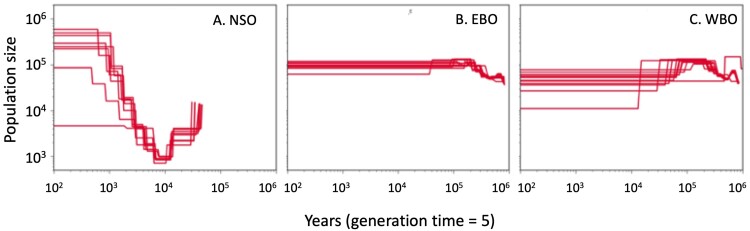
Demographic history inferred by SMC++ for (*A*) NSO, (*B*) EBO, and (*C*) WBOs. Each trajectory was drawn with different distinguished samples (Terhorst et al. 2017). A mutation rate of 4.6 × 10^−9^/bp/generation and a generation time of 5 years were used. The current day is all the way to the left on the *x* axis.

The longer scaffolds in our new assembly enabled us to estimate historical changes in population size (*N*_e_) using sequentially Markovian coalescent-based methods. We did this using SMC++ (Terhorst et al. 2017) for NSO, WBO, and EBO (fig. 3). Since SMC++ and related methods cannot accurately infer very recent changes in *N*_e_, we focused on the last 20–200,000 generations (0.1–1,000 ka assuming a generation time of 5 years). SMC++’s calculations require the specification of a “distinguished” individual from a population. We considered all possible distinguished individuals and plotted the estimated population size trajectories for each one as separate lines (fig. 3).

SMC++ inferred that the NSO population experienced a moderate population bottleneck down to *N*_e_ ∼ 1,000 roughly 2,000 generations ago (∼10 ka) (fig. 3*A*), followed by a population expansion to *N*_e_ of approximately 6 × 10^5^. On the other hand, the EBO and WBO populations never experienced any substantial reduction in population size (fig. 3*B* and *C*). Interestingly, the WBO population trajectories show some qualitative variability depending on the choice of distinguished individual. It is unclear how best to interpret this, but we suspect that it merely reflects random noise in the method.

### Split Time between BO Populations

If the WBO population split from EBO 80–130 years ago during the time of their documented migration westward (Livezey 2009a), then there are two main patterns that should be visible in patterns of genetic variation. First, WBO would have experienced a recent founder effect, leading to reduced variation and increased genetic drift. Second, genetic variation in WBO should be a subset of variation found in EBO, since there would not have been enough time for novel WBO-specific mutations to arise. We find evidence for a weak founder effect, including reduced diversity in WBO relative to EBO (supplementary table S8, Supplementary Material online), and a skew toward more common variants in WBO (measured by Tajima’s *D*, cf. supplementary table S10 and Supplementary Material, Supplementary Material online) as expected under a population bottleneck (Fay and Wu 1999). As both of these observations are not very strong, it is likely that any bottleneck that WBO experienced was neither severe nor recent.

**Fig. 4.**
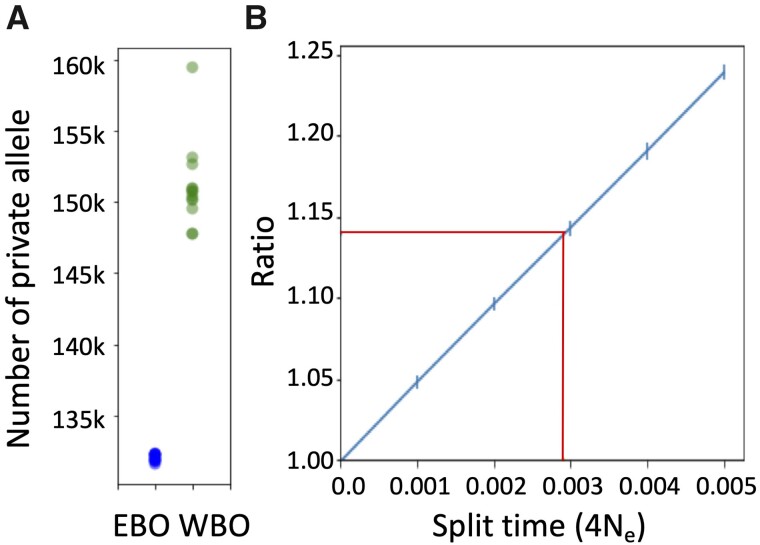
Estimation of split time between populations. (*A*) Observed numbers of private alleles of WBOs and EBOs were compared in 13 groups. A green dot shows the number of private alleles in each WBO sample, whereas a blue dot indicates the averaged number across the 12 EBO samples in a group. (*B*) Expected ratio of the number of private alleles in a WBO sample relative to the average number of private alleles across EBO samples plotted against split times between populations. Vertical blue bars show 95% confidential intervals and red line corresponds to the average ratio in the actual data.

To test whether the amount of WBO-specific genetic variation is in line with the expectation of a very recent split time, we tabulated the number of WBO-specific SNPs in each WBO sample (see Materials and Methods). We then compared this with a null model that assumes WBO and EBO samples came from the same panmictic population. We used this new approach because none of the commonly used methods to estimate split times of populations, such as SMC++ (Terhorst et al. 2017), ∂a∂i (Gutenkunst et al. 2009), and PSMC (Li and Durbin 2011), are able to estimate split times over such recent history (80–130 years ago). We also wanted to develop a method that is robust to the effects of population structure and/or bottlenecks following the population split. We found that WBO samples contained, on average, 14% more private variants than expected under the null model (fig. 4 and supplementary table S11, Supplementary Material online), and a simple permutation test shows this observation to be highly significant (*P* = 1.9 × 10^−7^). These results do not change if we exclude known functional regions (i.e., annotated exons) from our analyses (supplementary table S11*B*, Supplementary Material online). Assuming a simple divergence model, we estimate that EBO and WBO split from each other 0.0029 × 4*N*_e_ generations ago (fig. 4*B*). If we assume an average generation time of 5 years and an effective population size of 120,000, this corresponds to a divergence time of 7,000 years ago. Even if we assumed a 5-fold (total) uncertainty in the estimation of fundamental population genetic parameters, our divergence time estimate would range from 1.4 to 35 ka (see Supplementary Material, Parameters for population split time estimation for details, and supplementary table S12, Supplementary Material online), which is still well outside the commonly accepted 80–130 years ago range of Livezey (2009a). To get the divergence time estimate down to 140 years ago would require, for example, an average generation time of 3 years and a WBO mutation rate estimate of 1.34 × 10^−7^/site/generation, which is unrealistic since it would be several times higher than the highest estimated mutation rate in any eukaryote.

## Discussion

*Strix* owls have long been of great interest to many groups, partly because they are large, charismatic vertebrates, and partly because of the ecological, environmental, and economic consequences of listing NSO under the Endangered Species Act. Although there have been several genetic studies of SOs over the past 20 years (Barrowclough and Gutierrez 1990; Barrowclough et al. 1999, 2005, 2011; Haig et al. 2001; Haig, Mullins, Forsman, et al. 2004; Hanna, Henderson, Sellas, et al. 2017; Hanna, Henderson, Wall, et al. 2017; Hanna et al. 2018; Wojcik et al. 2019), there are still many unanswered questions related to population structure and hybridization. Our analysis of 51 high-coverage genomes is by far the largest genetic study of *Strix* owls, and the larger data set enabled us to conduct analyses that were not possible in earlier studies. For example, the longer scaffolds in our new genome assembly combined with high-coverage whole-genome sequence data enabled us to more accurately estimate past population sizes in both SO and BO (fig. 3), which showed that SO (but not BO) experienced a moderate population bottleneck that may have been coincident with the end of the last Ice Age.

Unexpectedly, we found substantial differentiation between WBO and EBO that is inconsistent with a separation time of between 80 and 130 years ago. There are two plausible explanations for this observation: First, we do not know “where” this divergence may have occurred. Given our limited sampling of EBO individuals, it is possible that there is a substantial amount of genetic variability within EBO, and that there exists an unsampled EBO population that is directly ancestral to extant WBO individuals. Work by Barrowclough et al. using mitochondrial data (Barrowclough et al. 2011) suggested that there is substantial variation within EBO. Although we confirmed that our samples included the population structure observed with mtDNA, it is still possible that our data do not fully cover the range of EBO diversity (Supplementary Materials, Supplementary Material online, mtDNA analyses). Based on figure 2*C*, we suspect that further sampling in or near Indiana may be fruitful in identifying a putative EBO “source” population for WBO. Second, it is possible that BOs were actually living in western North American forests before the earliest recorded observations. Our estimate of the population split time overlaps with the predicted time of recovery of forests that connected the eastern and western parts of North America after the last Ice Age (Adams 1997). Given this expansion of the possible habitats together with the weak signals of a founder effect in WBO, the migration of large numbers of BO to the new western habitat accompanying the recovery of the forests is a plausible explanation for the differentiation between WBO and EBO. It would not be very surprising if BOs, which maintained a large effective population size even during the last Ice Age, have lived for a long time in Canadian forests with cold temperatures. Additional BO sampling in both central North America and these northern forests will be crucial for distinguishing between the remaining hypotheses. Regardless, our data clearly refute a scenario in which the WBO samples are very recently derived (i.e., within the past 130 years) from a panmictic population of EBO (as encapsulated by the 12 EBO samples examined in this study). Since our analyses focused on BO-specific variants, we believe that this ambiguity in BO population history can only be explained by distinct evolutionary histories of the sampled WBO and EBO individuals. An older divergence time between WBO and EBO populations is also more consistent with the observed variability in EBO versus WBO plumage (cf. fig. 1, Hanna et al. 2018). Finally, we would like to emphasize that the results of our methodology are insensitive to unknown facets of WBO population history, such as any potential population bottleneck associated with the founding of WBO populations. This is because WBO demographic events that occur after the EBO–WBO split do not affect the distribution of coalescence times between EBO and WBO samples, nor the expected number of mutations on any WBO-specific branches of the genealogy.

As we explained above, we found that SO experienced a moderate bottleneck around the time of the end of the last Ice Age, followed by a recovery to even larger size of *N*_e_ than the one before the bottleneck. However, we did not observe a decrease in effective population size of NSO in the recent past on SMC++ plots though it is expected from the documentation of their recent decline in census size. We postulate that the decease is too recent (∼100 years ago) to be detected by SMC++ or Tajima’s *D* (supplementary table S10 and Supplementary Materials, Supplementary Material online), but that the reduced nucleotide diversity (supplementary table S8, Supplementary Material online) and number segregating sites (supplementary table S10, Supplementary Material online) emphasize a precarious future for SO.

SMC++ plots from WBO samples found no evidence for a bottleneck corresponding to the population split with EBO roughly 7,000 years ago. Instead, we observed variability in the trajectories, which probably reflects random noise in the method. This is broadly consistent with the *F*_ST_ and heterozygosity estimates, which showed that any bottleneck was likely to be weak and not very recent.

These findings on evolutionary and demographic history of BOs are also important for conservation of SOs since little was known about the history of their “invasive” species, BOs. So far, it has been believed that the habitat loss in the eastern part of the North America caused the migration of BOs to the west. But if BOs have lived in Canadian forests for a long time, their recent invasion to the range of SOs might be due to different reasons, such as the recent loss of boreal forests caused by the climate change and human activities (Gauthier et al. 2015).

Our use of whole-genome sequencing also allowed us to classify potential SO versus BO hybrid individuals. In contrast to a general lack of hybridization between NSO and WBO across much of their range (Hanna et al. 2018), hybridization appears to be a more significant phenomenon at the leading edge of the WBO expansion into regions such as the Northern Sierras where WBO are still rare (Kelly and Forsman 2004). However, as with our previous work (Hanna et al. 2018), we found that hybrid individuals are difficult to identify with certainty from physical appearance and morphological characteristics alone. Out of 15 potential hybrids in our sample, we identified four WBO, eight F1 hybrids, and three F1 × BO backcross individuals. In addition, we still have little understanding of the overall fitness and ultimate fates of hybrid individuals. In line with previous studies (Hamer and Forsman 1994; Kelly and Forsman 2004), we confirmed that there is a slight mating bias toward male SO × female BO. We also observed that the SO contribution to these hybrid individuals included NSO and F1 CSO × NSO individuals. Our results, however, cannot directly address the apparent absence of later-generation hybrids between SO and BO. We observed only three backcrosses, and all of them are backcrosses with WBO and are carrying BO mtDNA. Since we did not observe parent-offspring pair between F1 and a backcross in our samples (Supplementary Materials and supplementary fig. S15, Supplementary Material online), they could be either from matings between male F1 × female BO or between female F1 with BO mtDNA × male BO. According to Haldane’s rule, if only one sex is inviable or sterile in a species hybrid, that sex is more likely to be the heterogametic sex (in the case of birds, female). We could not conclude whether female hybrids can make the next generation or not from this data. It is also unclear at this time whether these later-generation hybrids are not found due to hybrid incompatibilities, or whether further sampling of potentially hybrid individuals would uncover a deeper collection of multigeneration hybrids. Additional in-depth studies of potential SO versus BO hybrids, along with a fully contiguous genome assembly, will be necessary to answer this question.

## Materials and Methods

### Assembly of the New Reference Genome

To obtain an improved SO reference genome, we generated a hybrid (10xG and Bionano Genomics) assembly following the approach in Levy-Sakin et al. (2019). Briefly, we obtained high-molecular-weight DNA from blood sample of Sequoia and used this to generate a 10xG linked-read library (using their Chromium system) and Bionano genome maps (using their Irys system). Instead of generating a single-genome map with the enzyme Nt.BspQI, we generated two sets of Bionano genome maps with the enzymes Nt.BspQI (New England Biolabs [NEB], Ipswich, MA, USA) and Nt.BbvCI (NEB, Ipswich, MA, USA). The 10xG library was sequenced to an average depth of approximately 60× and assembled using Supernova v1.1 (Weisenfeld et al. 2017). We then generated hybrid scaffolds using the Bionano genome maps to bridge Supernova scaffolds (see Levy-Sakin et al. 2019 for further details).

### Sequence Data

We utilized whole-genome sequencing data from a previous study (Hanna, Henderson, Wall, et al. 2017) for Sequoia (SRR4011595, SRR4011596, SRR4011597, SRR4011614, SRR4011615, SRR4011616, SRR4011617, SRR4011618, SRR4011619, and SRR4011620). For the other 50 samples from various sampling locations (fig. 1 and supplementary table S5, Supplementary Material online), we extracted genomic DNA following the method described in Hanna, Henderson, Wall, et al. (2017), prepared whole-genome libraries using a Nextera DNA Sample Preparation Kit (Illumina) and obtained high-coverage paired-end sequences from MedGenome, Inc. using a mix of Illumina HiSeq 2500 and 4000 machines. Samples were categorized into SO/BO/putative hybrids based on the morphology of the specimens and/or vocalizations. SO and BO samples were further categorized into CSO/NSO or EBO/WBO based on the sampling locations (supplementary table S5, Supplementary Material online). Two individuals were samples that had already been confirmed as hybrids in a previous study (Hanna et al. 2018). For convenience, we used simple informal IDs for all the samples, though corresponding museum IDs are shown in supplementary table S5, Supplementary Material online. The location map was made with leaflet package (Graul 2016).

### Alignment and Processing of Data

We processed the paired-end data from the whole-genome libraries of the 51 samples. We used Picard 2.19.0-SNAPSHOT in Genome Analysis Tool Kit (GATK) version 4.1.2.0 (McKenna et al. 2010; Depristo et al. 2011; Van der Auwera et al. 2013; Poplin et al. 2017) to remove adapter sequences. Then we modified the pipeline, processing-for-variant-discovery-gatk4.wdl supplied by the GATK as a Best Practice of GATK4, to use in our local environment. We aligned the trimmed paired reads to our new reference “StrOccCau_2.0_nuc_finalMito.fa” using bwa mem version 0.7.12-r1039 (Li 2013). We performed two rounds of base quality score recalibration in GATK4 using SNPs previously identified by Hanna, Henderson, Wall, et al. (2017).

### Variant Calling and Filtering

We called variants using the GATK4 HaplotypeCaller for each of the 51 samples and then performed joint genotype calling with the GATK4 GenotypeGVCFs tool for all samples included as simultaneous inputs. We used the GATK4 VariantFiltration to remove variants more extreme than a *P* value of 3.4e−6 in Hardy–Weinberg equilibrium, which corresponds to a phred-scaled value of 54.69.

We followed the guidelines of GATK for hard filtering (https://softw[are.broadinstitute.org/gatk/documentation/article?id=23216#2, https://software.broadinstitute.org/gatk/documentation/article?id=11069; last accessed April 5, 2021) to retain only high-quality, biallelic SNPs. First, we used the GATK SelectVariants tool to extract the SNPs from the raw VCF file. Then we filtered the SNPs using the GATK VariantFiltration tool with options “-filterExpression ‘QD < 2.0 ‖ FS > 60.0 ‖ MQ < 40.0 ‖ MQRankSum < −12.5 ‖ ReadPosRankSum < −8.0 ‖ SOR > 3.0’.” Then we removed any variants that fell within repetitive or low complexity regions using BEDTools version 2.25.0 (Quinlan and Hall 2010). To retain only biallelic sites, and to remove variants on the mitochondrial genome, we used the GATK SelectVariants tool with the “-restrict-alleles-to BIALLELIC -XL Sequoia_complete_mtGenome -exclude-filtered” options. We calculated the mean and standard deviation (SD) of the total unfiltered read depth across all samples per site, and removed all the variants exceeding the mean coverage plus five times the SD, as suggested by the GATK documentation. In addition to these basic filters, we filtered out individual variants with the minimum quality of assigned genotype (GQ) smaller than 40. We also removed the sites with missing data for all the analyses below except for the diversity analysis. For analyses of demography and genetic diversity, we removed four samples (ZRHG101, ZRHG123, ZRHG124, and ZRHG127) from the first degree relative pairs as described in the Supplementary Materials, Supplementary Material online.

### Sex Identification

A previous study (Hanna, Henderson, Wall, et al. 2017) identified scaffolds 806 and 4429 on their reference genome “StrOccCau_1.0_nuc.fa” as the scaffolds including matched sequences with CHD1Z or CHD1W, which are known as markers of sex for avian species (Fridolfsson and Ellegren 1999), suggesting that scaffolds 806 and 4429 are sequences from the Z and W chromosomes, respectively. We identified a corresponding scaffold for each of them in our reference genome “StrOccCau_2.0_nuc_finalMito.fa” with NCBI Blast and checked CHD1Z and CHD1W sequences were there. Using the difference in read depth on the correspondents, we identified sex for each of the 51 samples.

### Autosome Identification

Birds have the ZW sex-determination system, where the female is the heteromorphic sex (ZW) and the male homomorphic (ZZ). Since our reference genome is female, reads from both of the sex chromosomes were mapped to it. For identification of the Z chromosome and autosomes, we calculated the mean read depth for each scaffold in each sample. Then we took the averaged read depth of each scaffold across samples for males and females. Based on the assumption that the read depth of the Z chromosome would be half in females as in males, we searched for scaffolds with approximately half the averaged read depth across variants in female samples as in male samples, and identified them as sequences that likely map to the Z chromosome. We also identified the scaffolds with similar read depth in males and females as autosomes.

For identification of the W chromosome, we quantified the amount of missing data, because in males the variants on the W chromosome should be missing. To exclude low-quality regions, we applied a GQ filter of ≥40 (using vcftools, Danecek et al. 2011) and removed variants where more than half of the samples had missing genotypes. (Note that 26 out of the 51 samples are female.) For the final set of variants, we calculated percentages of missing data for each scaffold and contig of each sample. We searched for scaffolds or contigs where more than 99% of sites are missing in all male individuals in the pool of scaffolds and contigs longer than 100 kb, identifying them as W chromosome sequences. We used the autosomes only for the analyses.

### Principal Component Analyses

For PCA analysis, we pruned variants to leave variants with minor allele frequency at least 1%, with no pairs remaining with *r*^2^ > 0.2 for the sets of samples, using PLINK (Purcell et al. 2007). Then we performed PCA with PLINK.

### Identification of Close Relatives

We sought to identify closely related individuals in order to avoid possible nonindependence of close relatives or other effects of related individuals on our analyses of demography and genetic diversity. Since we do not have phased haplotypes for the sequenced genomes, we could not use standard identity-by-descent methods for detecting close relative pairs. Instead, we calculated the kinship coefficient (phi) (Manichaikul et al. 2010) and proportion of the sites where two individuals share zero alleles identical by descent (proportion of zero IBS) for each pair of individuals (see Supplementary Materials, Supplementary Material online, for further details).

### Diversity Analyses

For diversity analyses, we converted all variants with GQ < 40 to missing data with vcflib (Garrison 2021) for each individual. We calculated the number of segregating sites and Tajima’s *D* (Tajima 1983) for each population with vcftools (Danecek et al. 2011). We calculated *F*_ST_ with PLINK (Weir and Cockerham 1984; Purcell et al. 2007) and nucleotide diversity within and between populations or groups (Nei 1987) using custom python scripts.

### Hybrid Characterization

To estimate the percentage of SO ancestry in hybrids, we identified apparent fixed differences between 11 SOs and 25 morphologically identified BOs in our samples. For each known or potential hybrid, we calculated the mean percentage of ‘SO alleles’ at these fixed differences as well as the mean heterozygosity.

Similarly, we identified apparent fixed differences between NSO and CSO, at sites where no polymorphism is observed in BO samples, to estimate the percentages of subspecific spotted owl ancestries in hybrids. Assuming one of the parents of each hybrid is a BO, we tabulated the mean percentages of “NSO alleles” across these NSO versus CSO fixed differences for each hybrid individual.

### Generation Time for Analyses

Estimates of the mean generation time in SOs range from two (Gutiérrez and Franklin 1995) to five (Barrowclough and Coats 1985; Barrowclough et al. 1999) or 10 years (Noon and Biles 1990; USDA Forest Service 1992). When we considered the reported low rate of successful breeding in young adult SOs (Forsman et al. 2002), 5–10 years seems to be reasonable. We used a mean generation time of 5 years to be conservative relative to split time estimates (i.e., to err on the side of underestimating the true split time).

### Inference of Population Size Histories

To estimate population size and infer demographic histories for NSOs, EBOs, and WBOs, we used SMC++ version 1.15.2 (Terhorst et al. 2017). We did not specify time points option to use a heuristic to calculate the model time points. We applied the mutation rate of collared flycatcher (*Ficedula albicollis*), 4.6e−9/site/generation (Smeds et al. 2016) and a generation time of 5 years (see above). Because it is known to be difficult to infer very recent changes in *N*_e_, we focused on the last 20–200,000 generations (100–1,000,000 years ago with a generation time of 5 years) for NSO, WBO, and EBO. We estimated the population size history multiple times using each different sample in a population as the distinguished individual, which is required in SMC++.

### Inference of Divergence Time between BO Populations

To infer whether WBO have been evolutionarily isolated from EBO for a substantial amount of time, we tabulated the numbers of “private” alleles in each WBO sample not present in EBO. Specifically, we considered all possible groups of 13 BOs consisting of 1 WBO and all 12 EBO samples; for each such group, we tabulated the total number private alleles for each of the 13 samples. Private alleles here mean alleles present in one sample but not the other 12 (i.e., singletons + 2 × private homozygotes). Standard coalescent theory predicts that the number of private alleles in a particular sample is roughly proportional to the mean coalescent time between the particular sample and any of the remaining 12 samples in the group. If WBO diverged very recently from a panmictic EBO population, then the number of private alleles specific to WBO samples should be roughly the same as the number of private alleles in EBO samples (averaged across samples and across groups). In contrast, if WBO populations have been genetically isolated from EBO populations for thousands of years, this would result in longer coalescent times between WBO and EBO samples, which in turn would lead to more private alleles for the WBO samples in the groups described above (supplementary fig. S10, Supplementary Material online). The asymmetrical definition of groups makes the mutations that have accumulated in WBO since the population split from EBO visible as private alleles in a single WBO sample in a group and makes the results insensitive to unknown facets of recent WBO population history, such as a population bottleneck. The effect of population structure within source, EBO population is also mitigated here, because we count only private alleles for individuals and do not use alleles with intermediate frequency, which are related to population structure. Counting not only singletons but also private homozygotes makes this method robust against high background level of inbreeding.

Under this framework, it is straightforward to estimate population divergence times using a simple split model. We assume our EBO samples are from a panmictic population, and that WBO form a separate panmictic population that separated T generations ago. We then run coalescent simulations (Hudson 2002) over different values of T and tabulate the expected ratio in the number of WBO private alleles versus EBO private alleles in the groups of 13 BO described previously. We then use a simple moment estimator (with linear interpolation) to estimate T from observed private allele counts. Note that these calculations are not affected by any changes in population size (e.g., population bottlenecks) experienced by WBO subsequent to their split from EBO.

For computational convenience, we simulated 100,000 short segments of 10 kb to mimic an entire genome of 1 Gb. Further, we assumed a mutation rate of 4.6 × 10^−9^/bp/generation (Smeds et al. 2016), an effective population size of 120,000 (estimated from the nucleotide diversity found within BOs) and a recombination rate equal to the mutation rate. We simulated 100 total whole-genome-equivalent replicates and counted private alleles in a group of one simulated WBO and 12 simulated EBO in exactly the same way we did for the observed data. To convert coalescent time units into years, we assumed a mean generation time of 5 years (see above).

Finally, to explore whether the inclusion of regions subject to natural selection biased our estimates, we reran our analyses after removing annotated exon sequences from the data. We utilized the gene annotation file provided by Hanna, Henderson, Wall, et al. (2017). We generated chain files for converting coordinates from StrOccCau_1.0 (Hanna, Henderson, Wall, et al. 2017) to our new assembly, StrOccCau_2.0, following the protocol described at the UCSC Genome Browser (http://genomewiki.ucsc.edu/index.php/Minimal_Steps_ For_LiftOver; last accessed April 5, 2021), then used liftOver (Hinrichs et al. 2006) to convert coordinates in the annotation file.

## Supplementary Material

Supplementary data are available at *Genome Biology and Evolution* online.

## Supplementary Material

evab066_Supplementary_Data
