# Supplementary material for: Genomic Variation and Recent Population Histories of Spotted (Strix occidentalis) and Barred (Strix varia) Owls
Source: Genome Biol Evol. 2021 Mar 25;13(5):evab066. doi: 10.1093/gbe/evab066 (PMC8120011; doi:10.1093/gbe/evab066)

Supplementary figures

Fig. S1 Comparison of the distribution of the lengths of scaffolds and contigs between the assemblies.

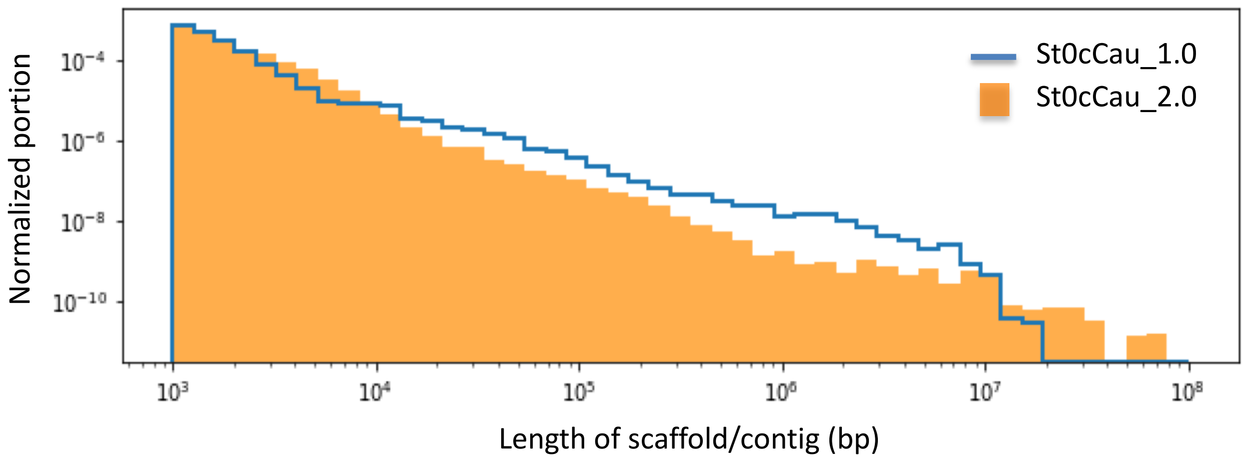

Fig. S2 Histogram of the mean read depth of scaffolds (>= 1 Mb) in males and females.

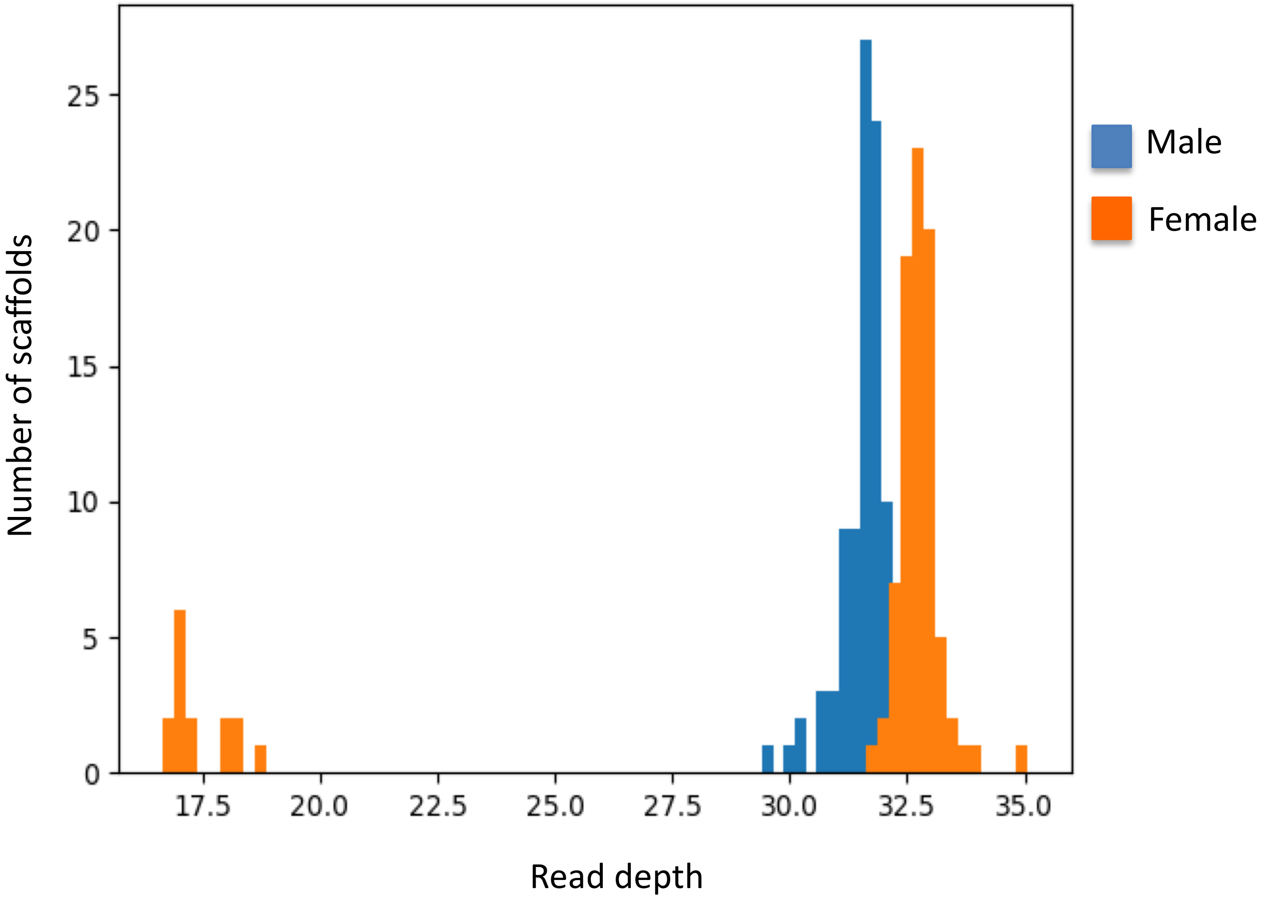

Fig. S3 Histogram of the proportion of missing data in scaffolds and contigs (>= 100kb, < 1Mb) in males and females.

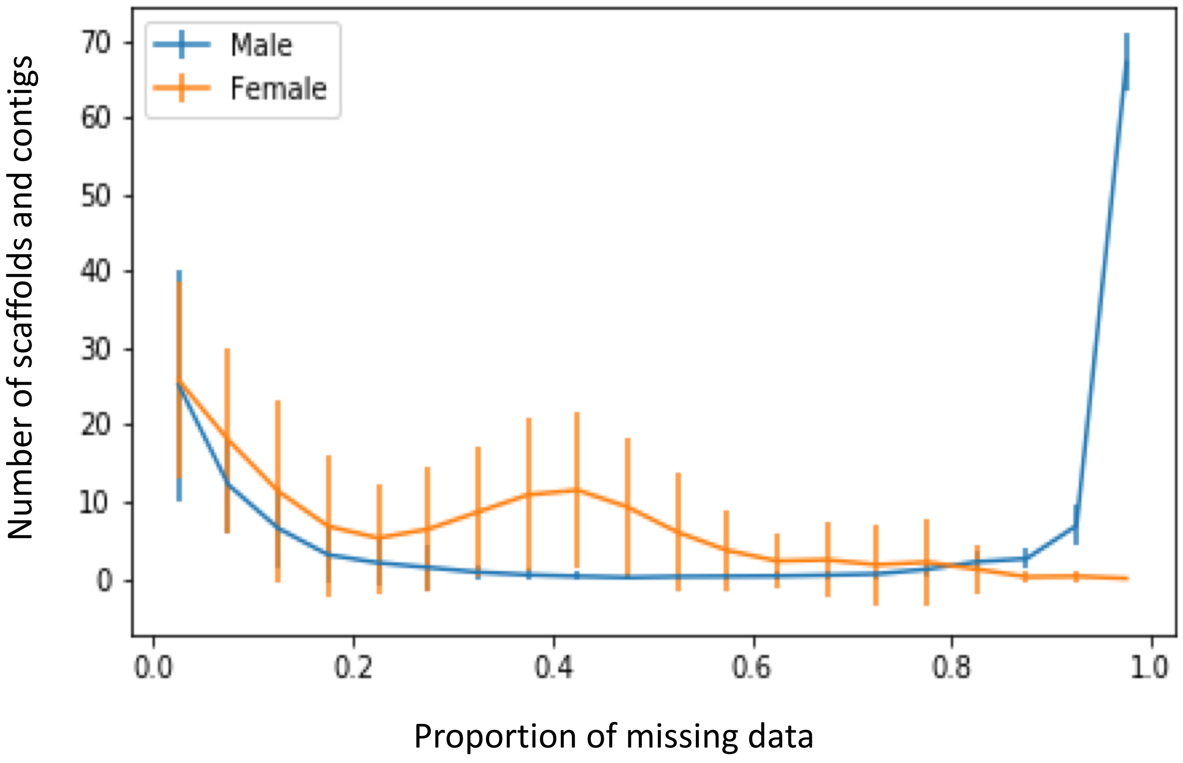

Fig. S4

Description of variants identified on autosomes and sex chromosomes.

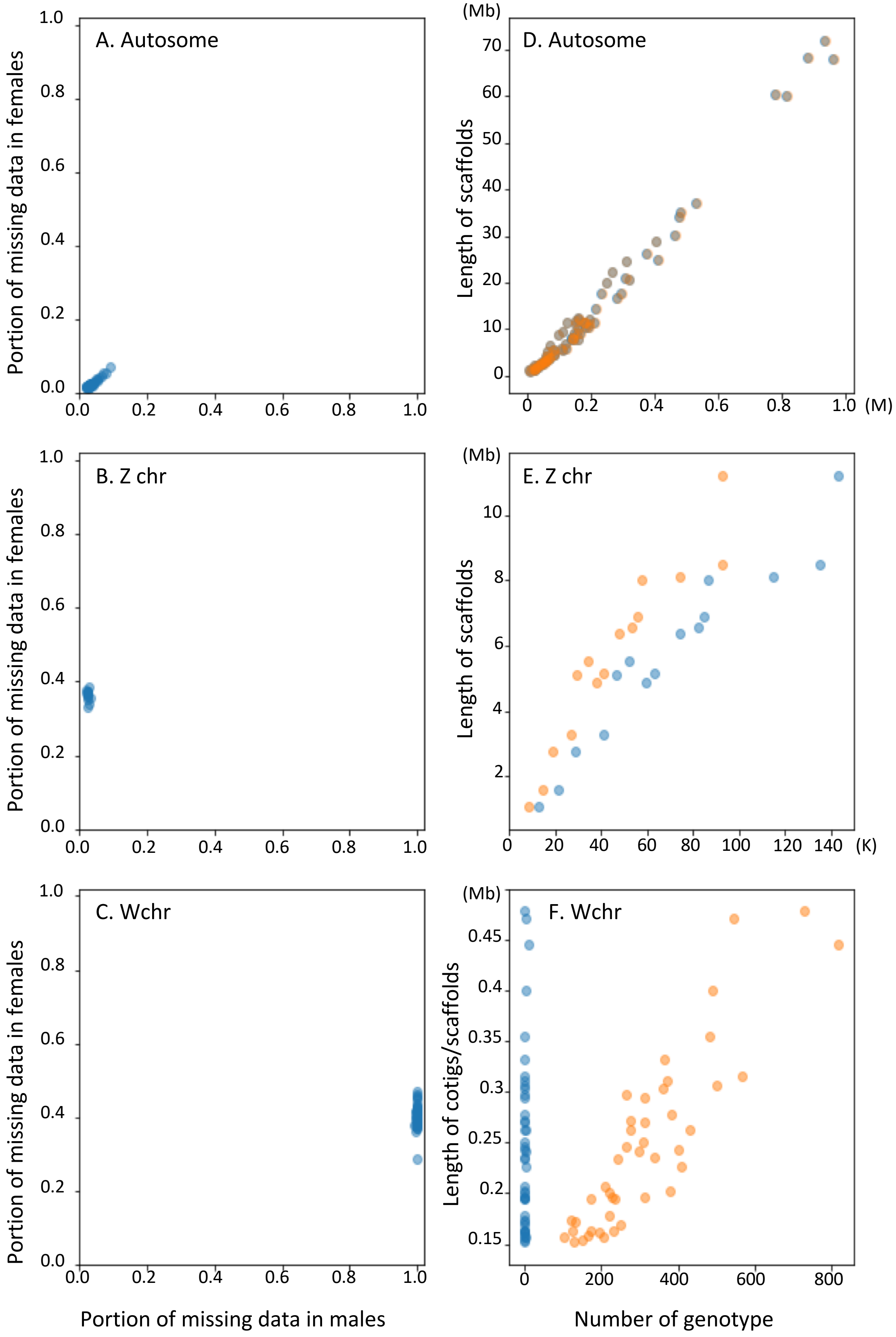

Fig. S5   Sampling locations of genetically identified samples

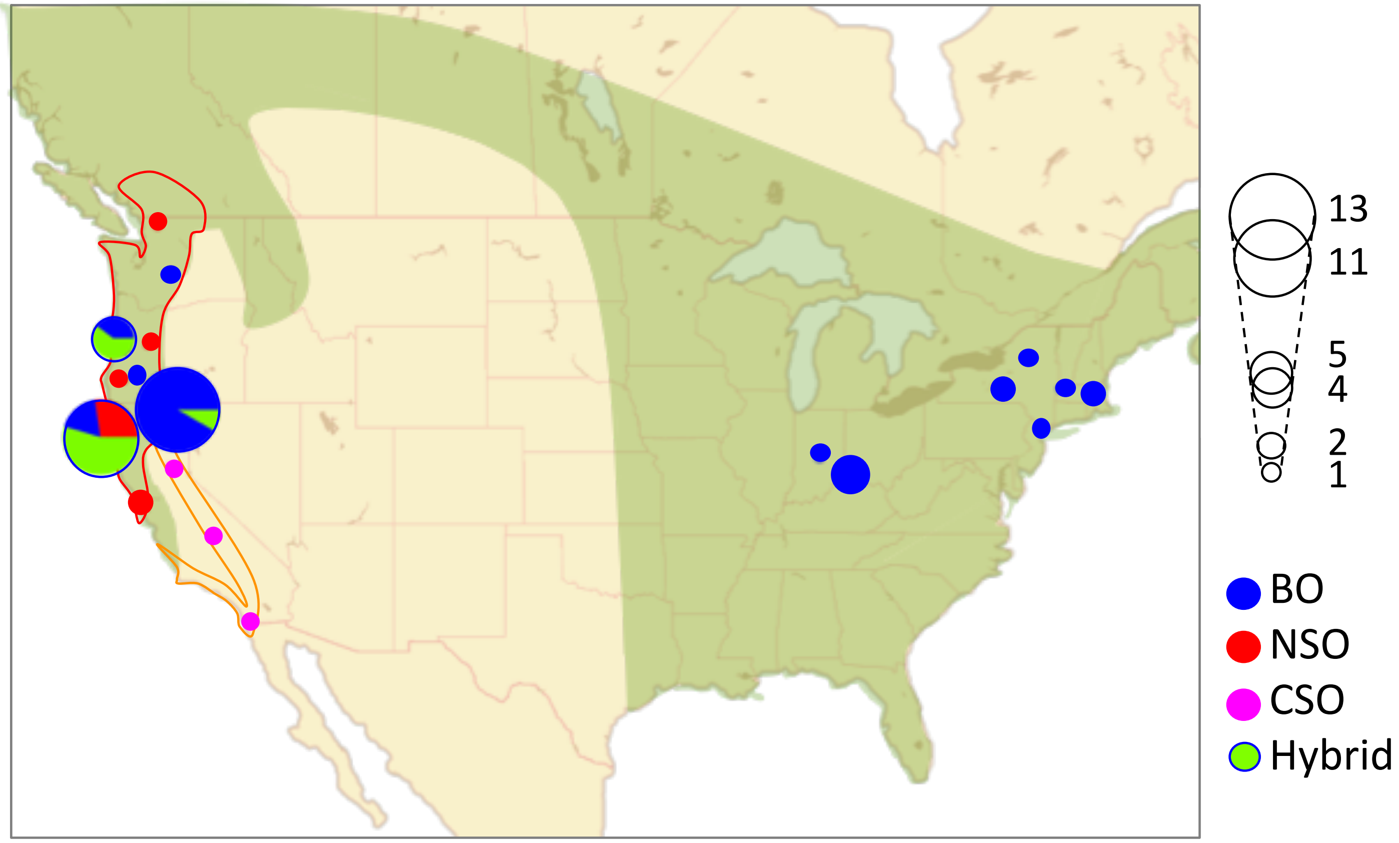

Fig. S6 PCA plot of Barred Owls.

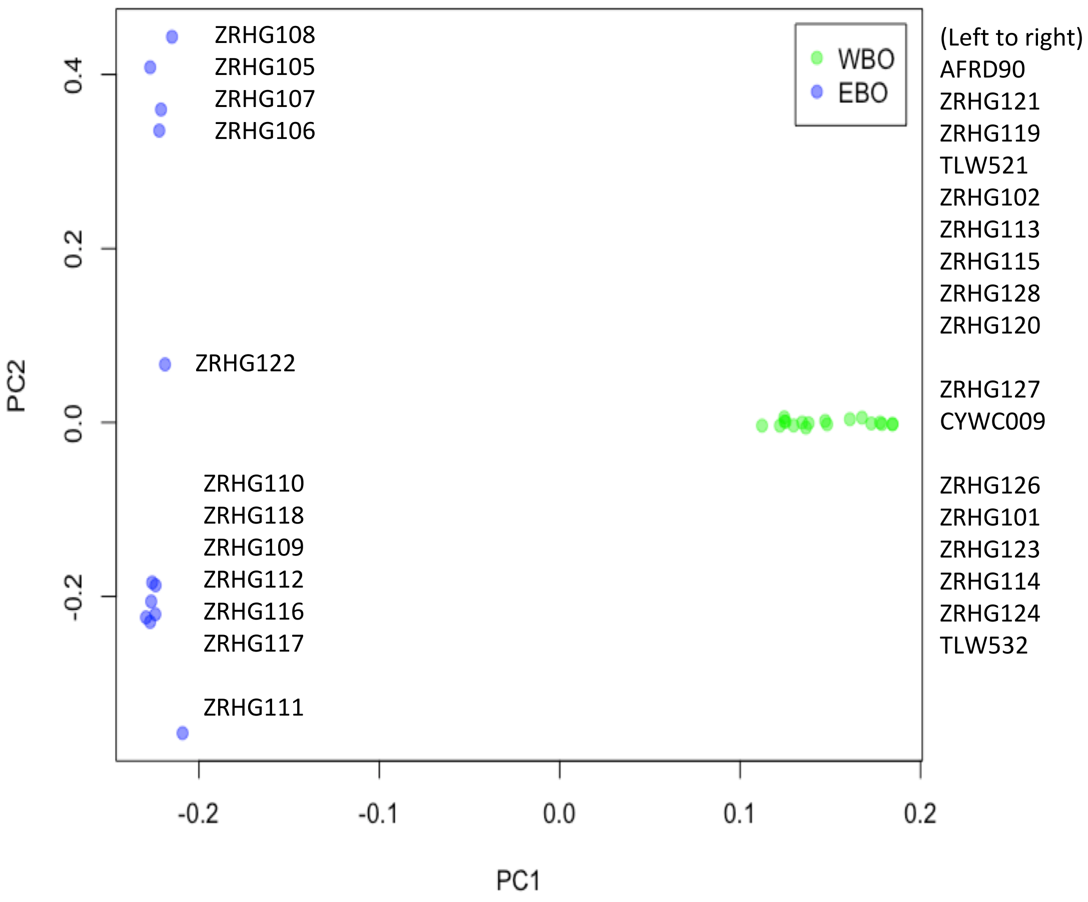

Fig. S7 Nucleotide diversity between EBO and WBO

(A) Pi between a EBO sample and WBO samples.

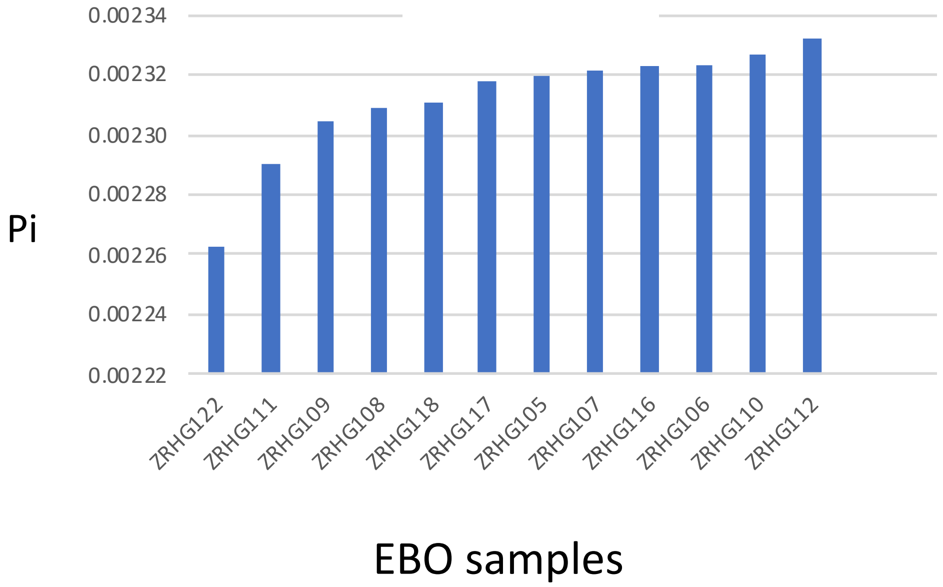

(B) Pi between a WBO sample and EBO samples.

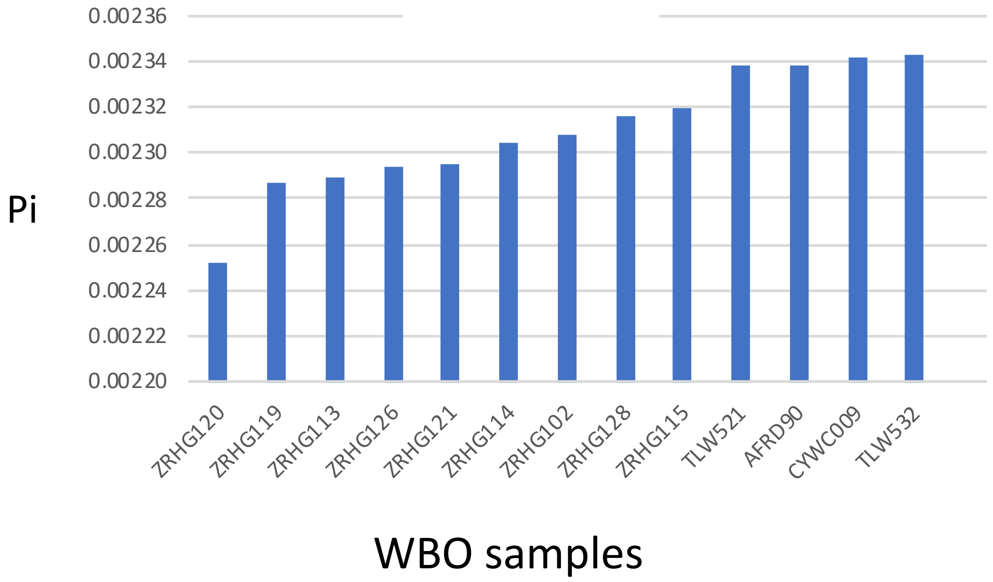

Fig. S8   ADMIXTURE analysis

(A) Cross-validation errors with different K values.

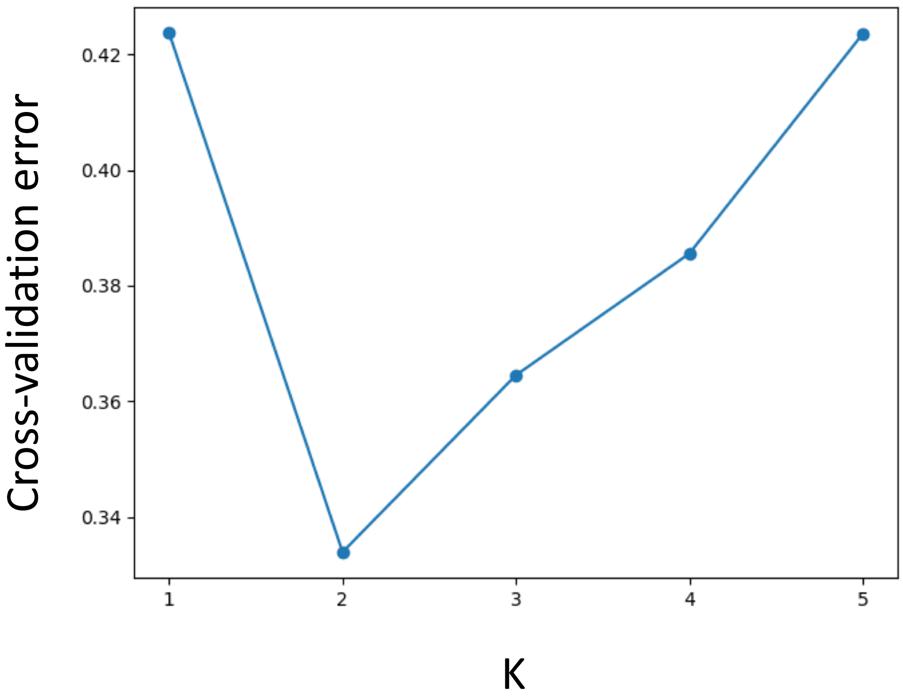

(B) ADMIXTURE plots under models
with different number of ancestral populations.

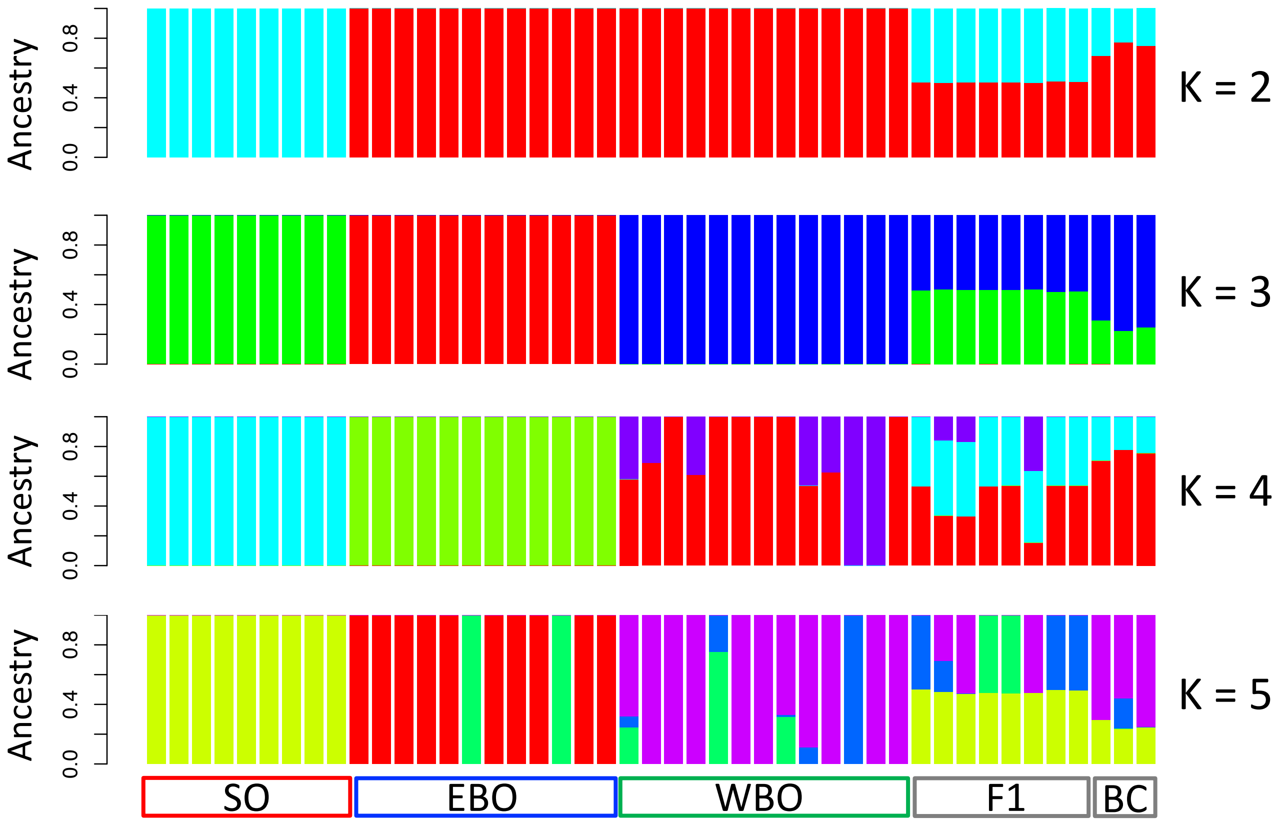

Fig. S8 ADMIXTURE analysis

(C) Cross-validation errors with different K values for SO.

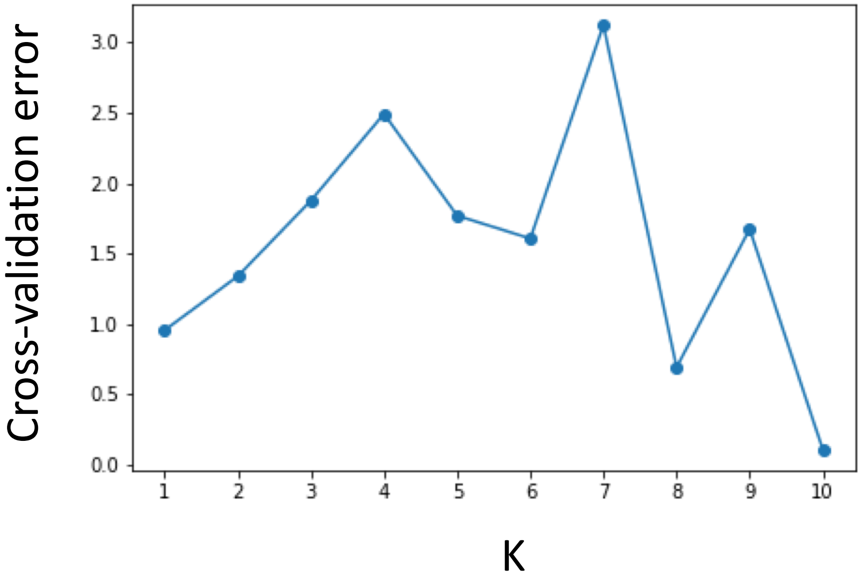

(D) ADMIXTURE plots for SO under models with different number of ancestral populations.

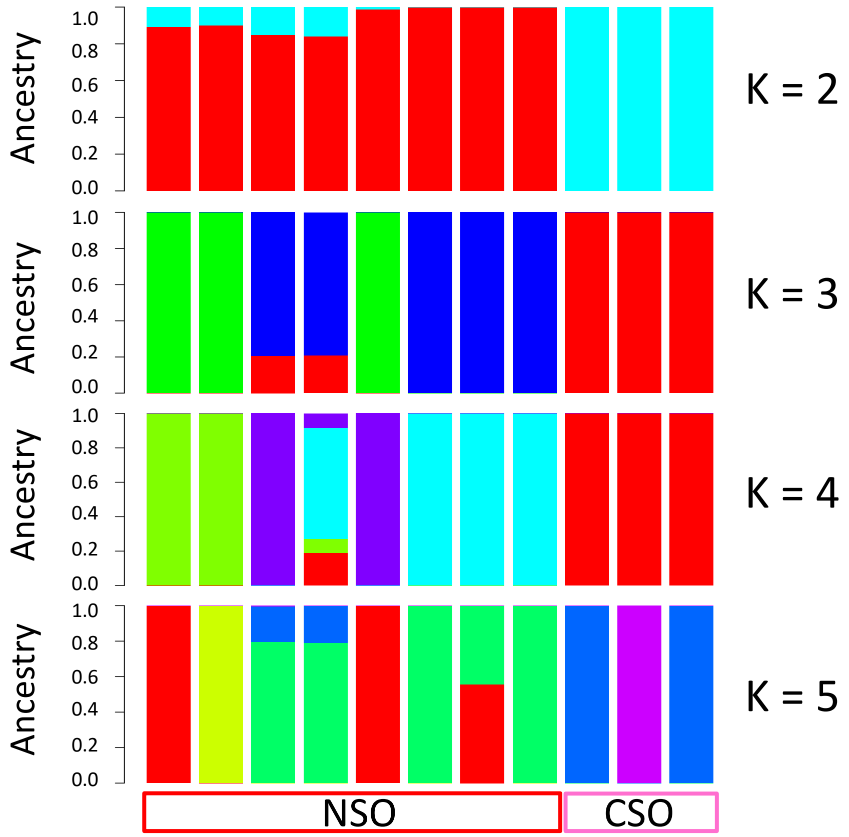

Fig. S9 The phylogenetic tree on the non-control region of mtDNA.

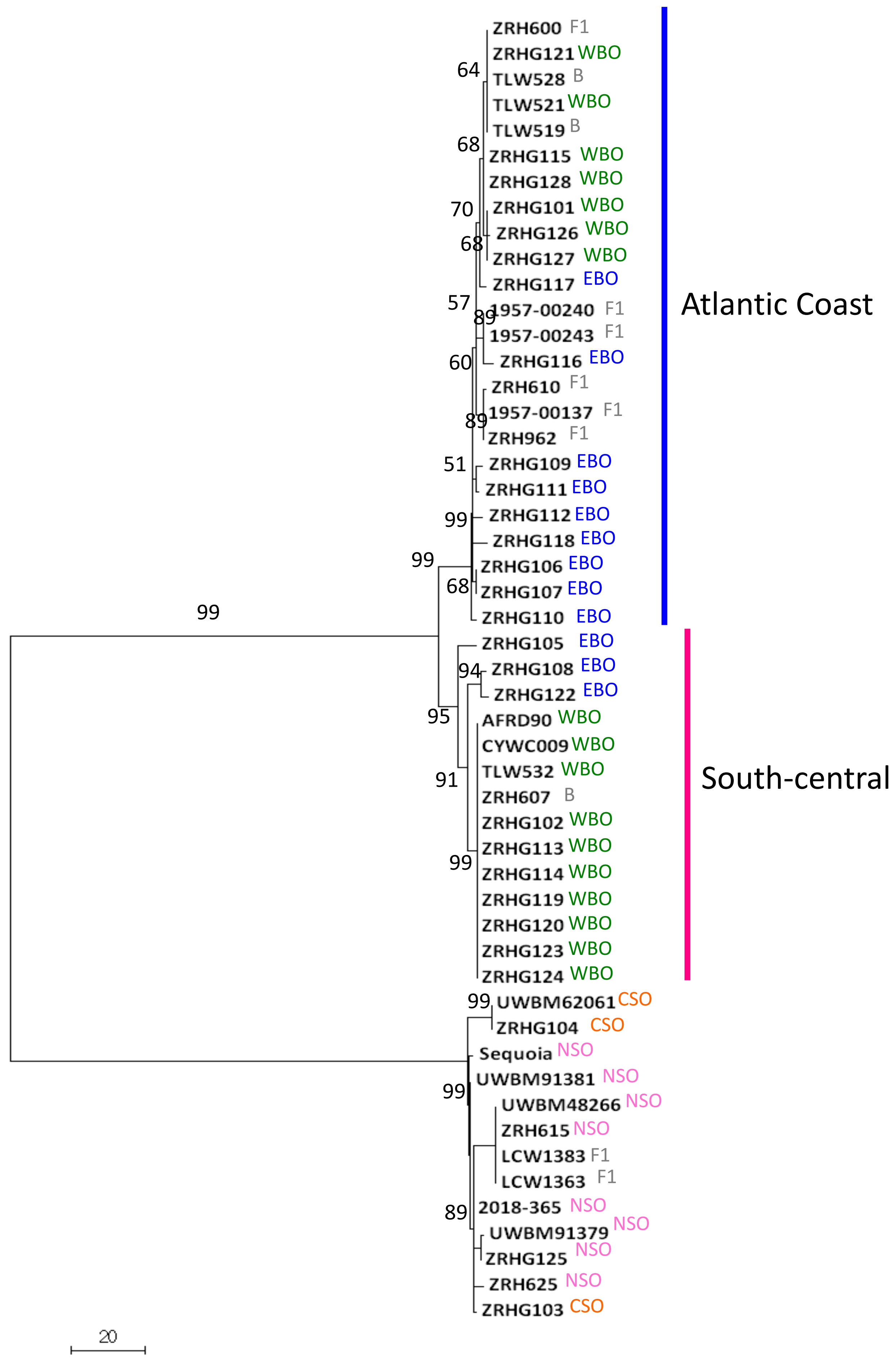

Fig. S10 Schematic picture of private alleles and population structure.

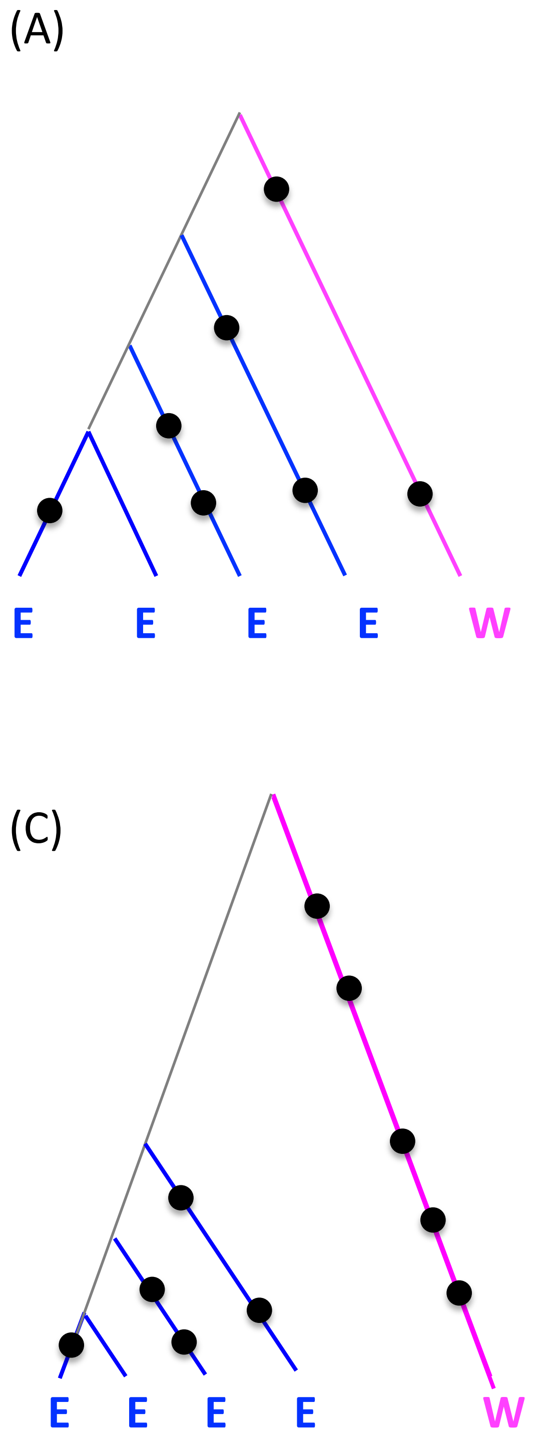

Fig. S11 Comparison of the two estimators of the kinship coefficient.

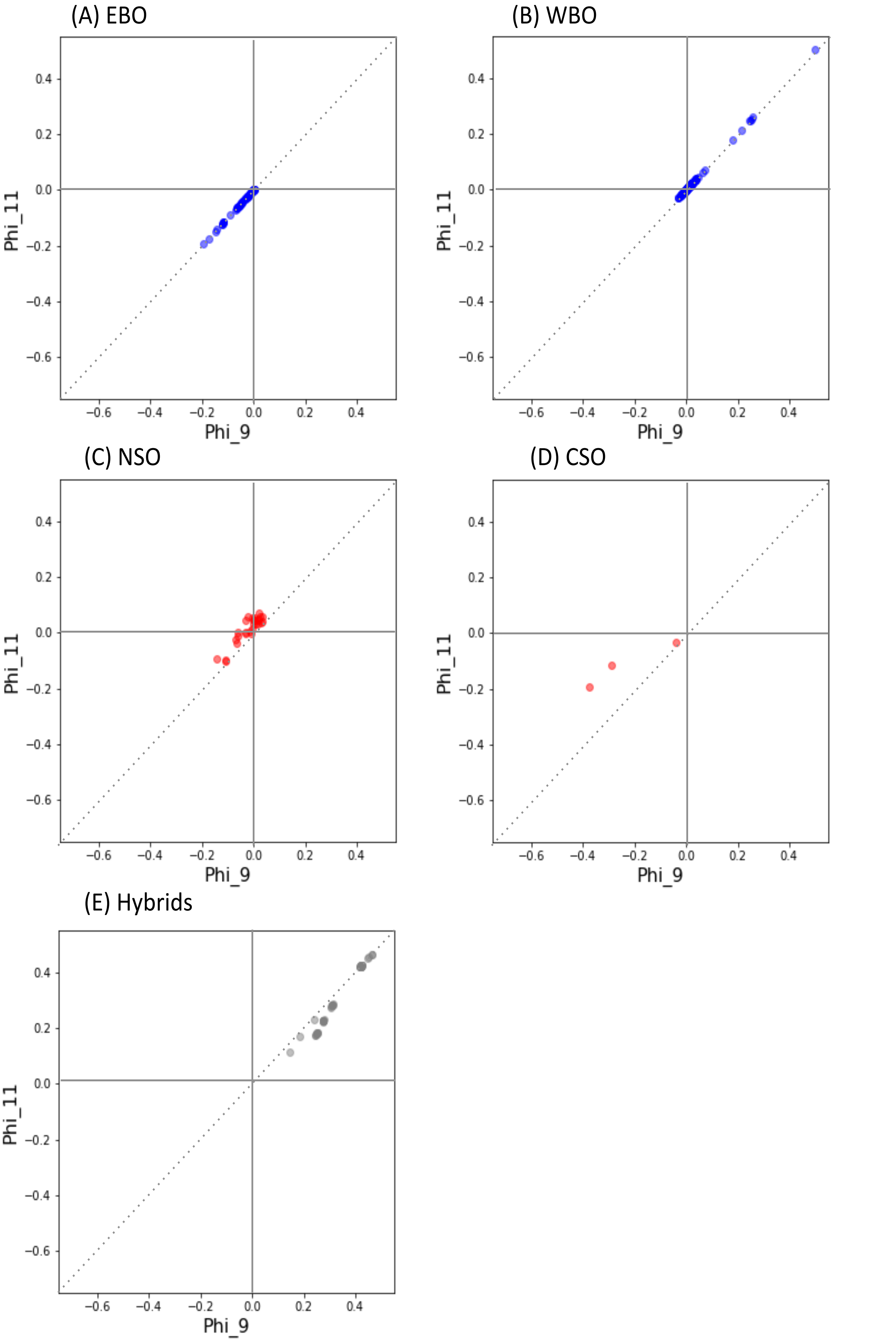

Fig. S12 Inference of related individuals within populations.

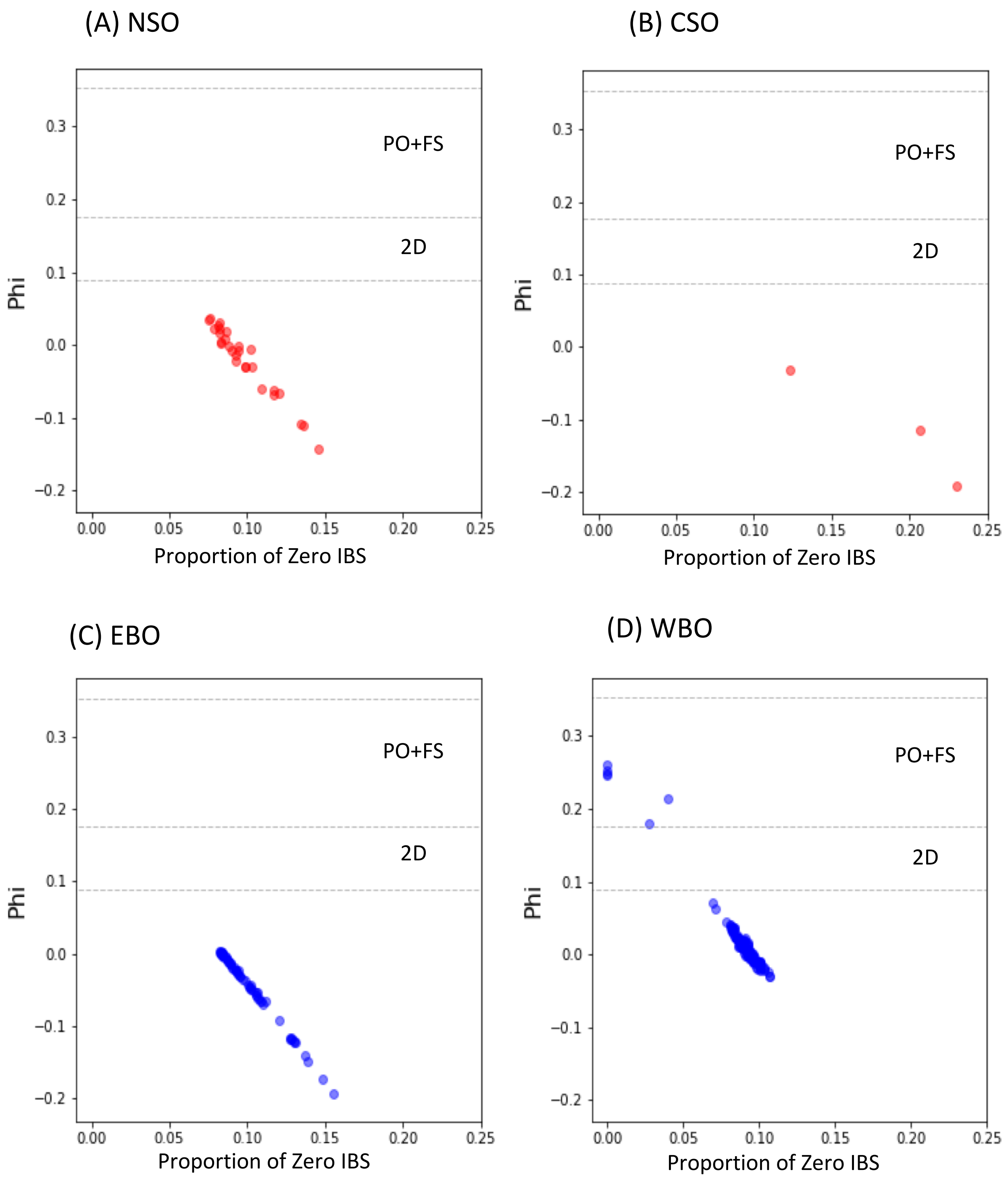

Fig. S13 Inference of related individuals between populations.

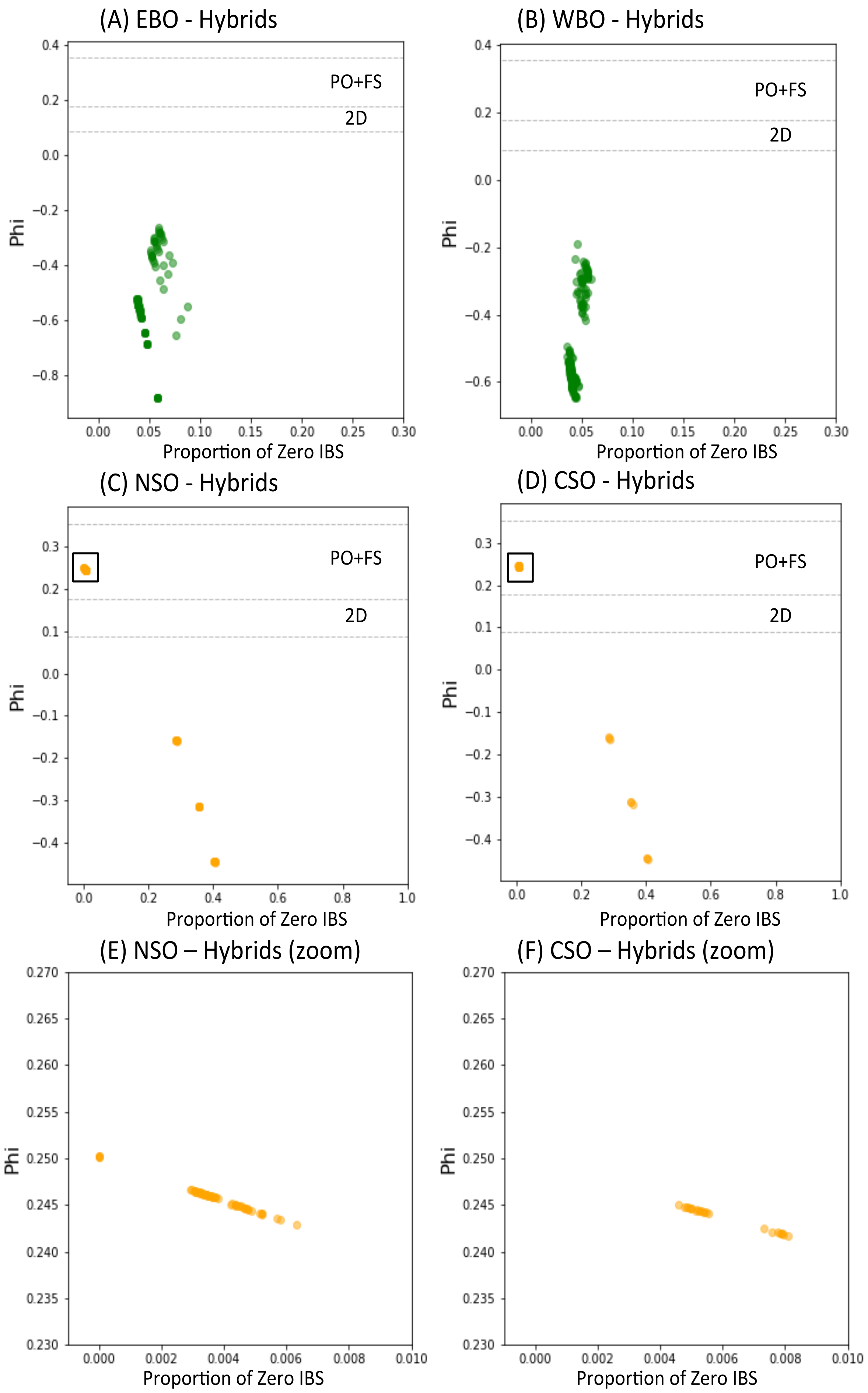

Fig.S14 Sampling location, number of segregating sites and number of zero IBS sites for the pairs with high phi values.

ID	Category1	Category2	Sampling location	
			County	State
Sequoia	SO	NSO	Marin County	California
2018-365	SO	NSO	Marin County	California
ZRHG125	SO	NSO	Humboldt County	California
ZRH615	SO	NSO	Humboldt County	California
ZRH625	SO	NSO	Humboldt County	California
UWBM48266	SO	NSO	Whatcom County	Washington
UWBM91379	SO	NSO	Linn County	Oregon
UWBM91381	SO	NSO	Baker County	Oregon
ZRHG103	SO	CSO	Nevada County	California
ZRHG104	SO	CSO	San Diego County	California
UWBM62061	SO	CSO	Tulare County	California
1957-00137	Hybrid	F1	Humboldt County	California
1957-00240	Hybrid	F1	Humboldt County	California
1957-00243	Hybrid	F1	Humboldt County	California
ZRH600	Hybrid	F1	Humboldt County	California
ZRH610	Hybrid	F1	Humboldt County	California
ZRH962	Hybrid	F1	Humboldt County	California
LCW1363	Hybrid	F1	Shasta County	California
LCW1383	Hybrid	F1	Siskiyou County	California
TLW519	Hybrid	Backcross	Lane County	Oregon
TLW528	Hybrid	Backcross	Benton County	Oregon
ZRH607	Hybrid	Backcross	Benton County	Oregon

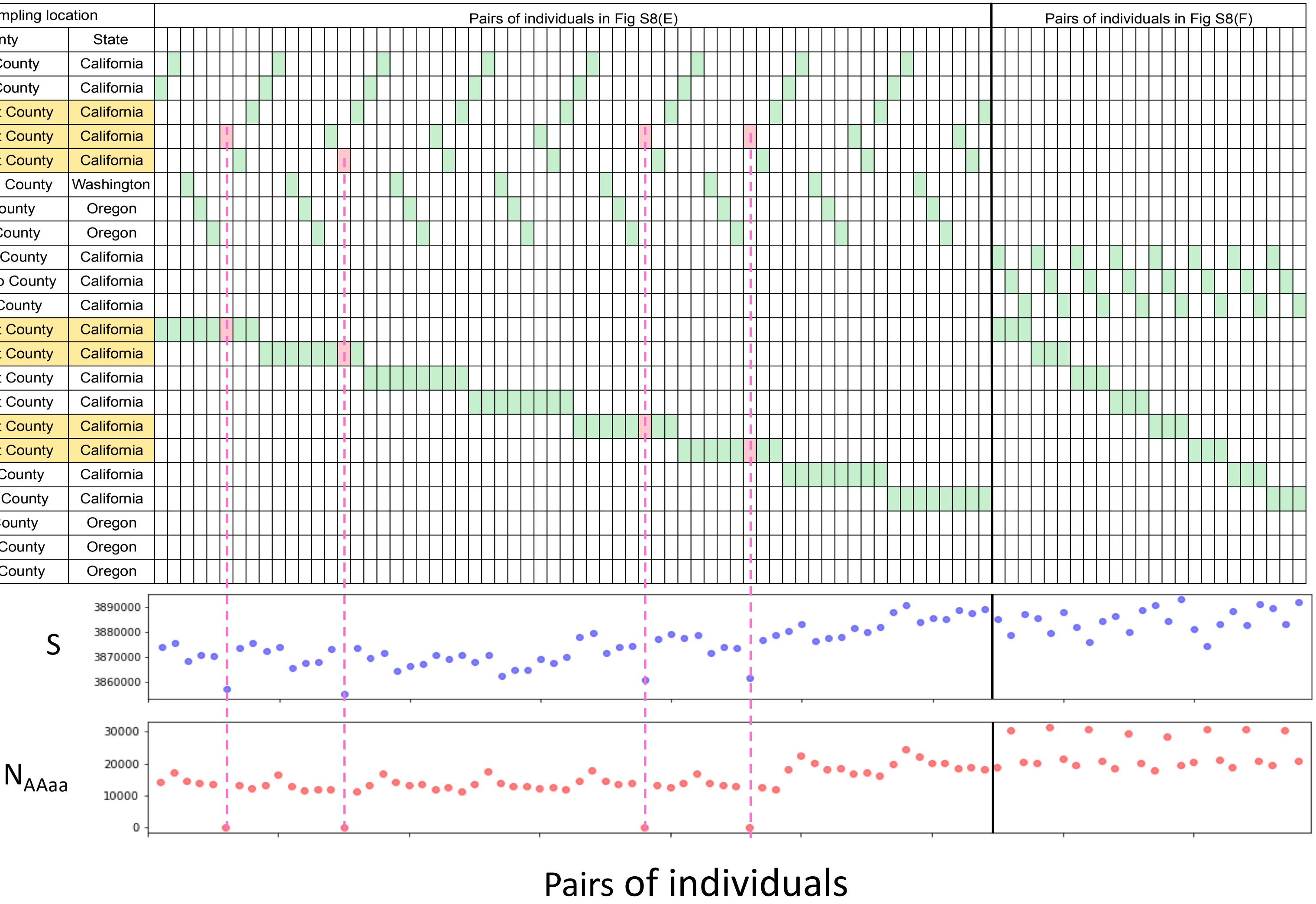

Fig.S15 Number of zero IBS sites and segregating sites for the pairs within F1 and between F1 and a backcross.

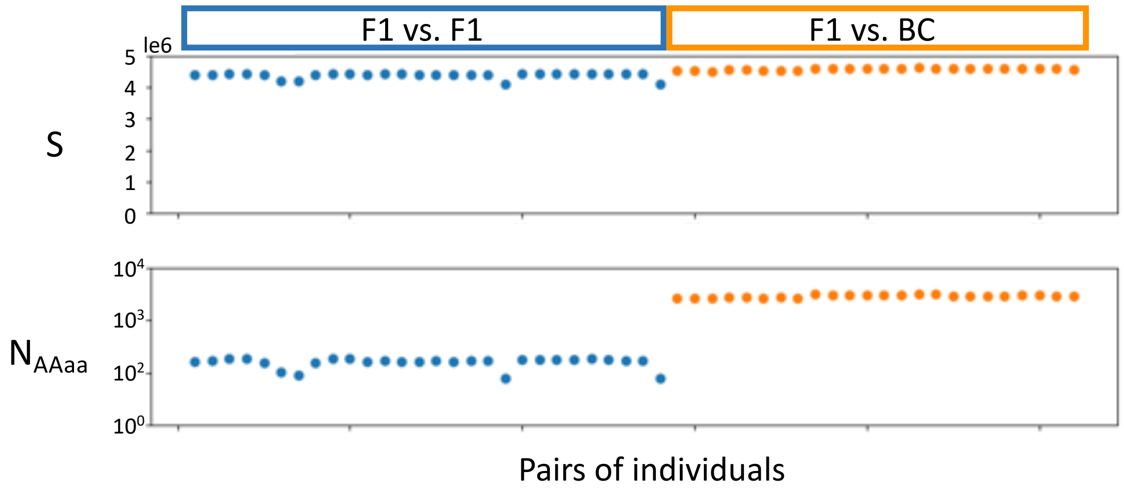

Fig. S16 The mean DP and the number of missing individual data for variants on mtDNA.

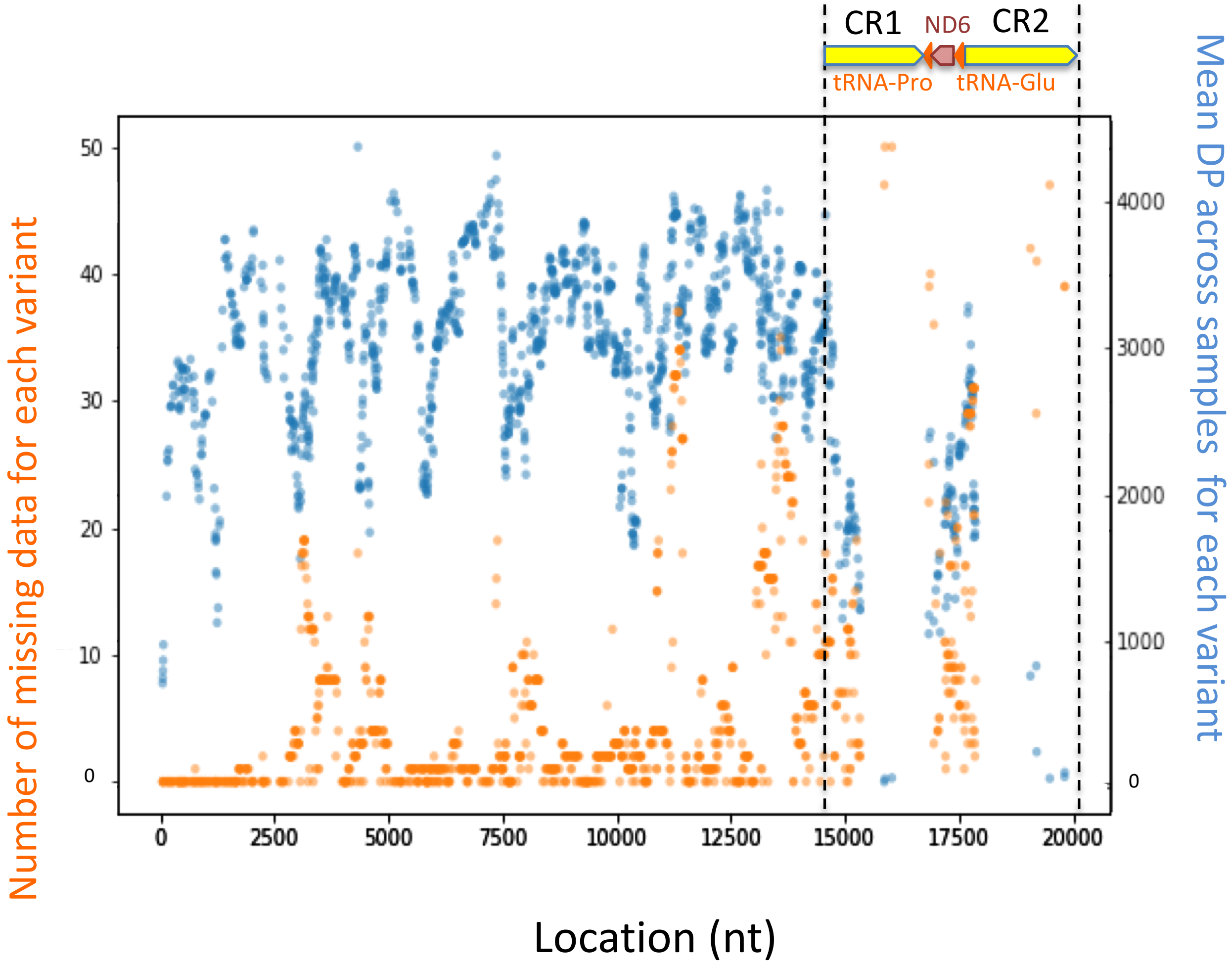

Fig. S17 Geographic distribution of the mitochondrial haplotypes.

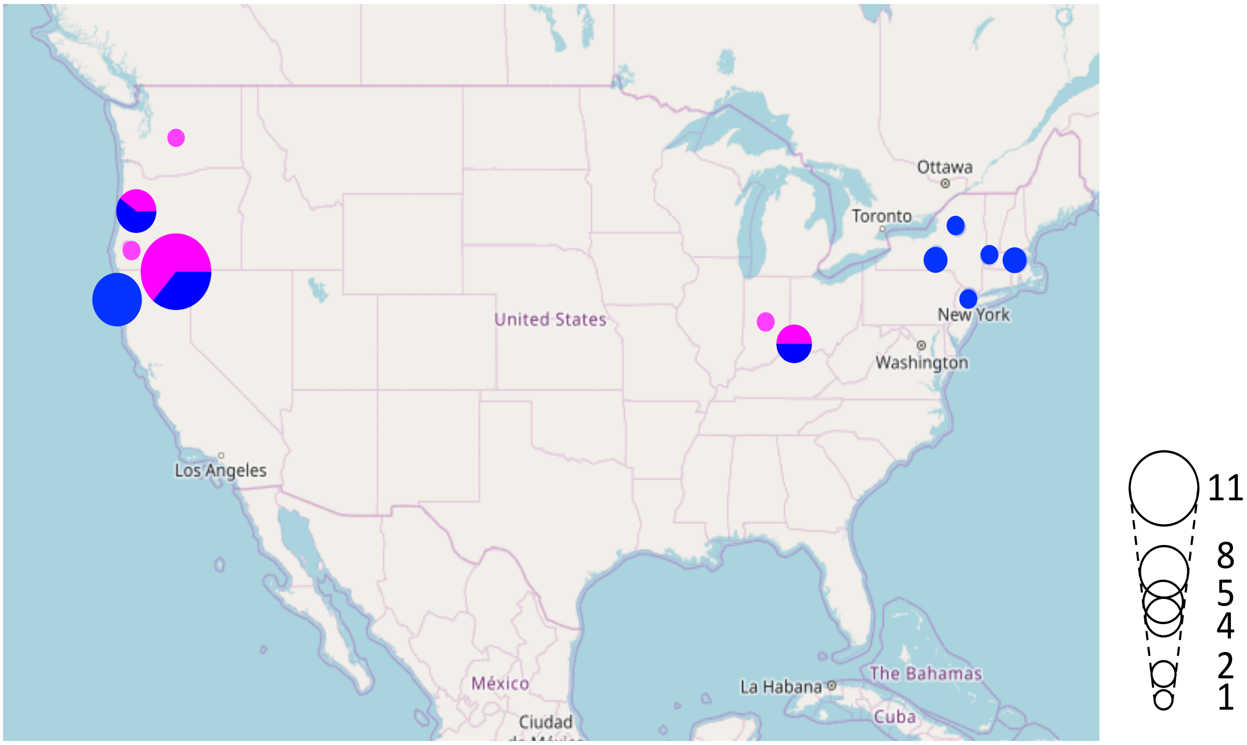

Supplement: evab066_Supplementary_Data [file evab066_supplementary_data.zip › SupFig_201201.pdf]
