# Supplementary material for: Genomic Variation and Recent Population Histories of Spotted (Strix occidentalis) and Barred (Strix varia) Owls
Source: Genome Biol Evol. 2021 Mar 25;13(5):evab066. doi: 10.1093/gbe/evab066 (PMC8120011; doi:10.1093/gbe/evab066)
Supplement: evab066_Supplementary_Data [file evab066_supplementary_data.zip › Supplementary_materials_201027_210103_210314.docx]

**Supplementary Materials**

1. Identification of close relatives

2. ADMIXTURE analysis

3. Tajima’s D for populations

4. mtDNA analyses

4-1. Extraction of mitochondrial variants

4-2. Phylogenetic analysis of the mitochondrial non-coding region

4-3. Identification of mitochondrial lineages

5. Parameters for population split time estimation

Captions for Supplementary Figures

Captions for Supplementary Tables

Supplementary reference

Supplementary figures

Supplementary tables

**1. Identification of close relatives**

We sought to identify closely related individuals within species/population in order to avoid possible non-independence of close relatives or other effects of related individuals on our analyses of demography and genetic diversity. We also identified related pairs of samples between purebread individuals and hybrids to record full relatedness among specimens for future studies. Since we do not have phased haplotypes for the sequenced genomes, we could not use standard Identity-By-Descent (IBD) methods for detecting close relative pairs. Instead, we used Identity-By-State (IBS) calculations as follows. We calculated kinship coefficient (phi) (Manichaikul et al. 2010) and proportion of the sites where two individuals share zero alleles (proportion of zero IBS sites, IBS0) for each pair of samples within and between populations. We used the proportion of zero IBS sites to distinguish parent-offspring pairs and pairs of full siblings, since the expected phi values of these two categories are the same, 0.25. The expectation of IBS0 for parent-offspring pairs is always 0, while that of full siblings cannot be 0. Combining phi and IBS0, we detected closely related samples (within 2^nd^ degree relatives) and estimated their relationship.

We used the “relatedness2” option in vcftools(Danecek et al. 2011) to calculate statistics related to phi and to count the total number of sites where no alleles are shared between the two individuals of a pair. Using the statistics from vcftools, we calculated an estimator of phi, with Equation (11) in Manichaikul et al. 2010(Manichaikul et al. 2010). Originally they use Equation (9) for within-family relationship checking and Equation (11) for between-family relationship checking. Because Hardy-Weinberg Equilibrium (HWE) among SNPs is assumed for Equation (9), Equation (11) was derived in order to guard against potential estimation inflation due to departure from individual-level HWE. If there is no departure from individual-level HWE, the estimator of Equation (9) is no larger than that of Equation (11). We used Equation (11) for our data, because we observed departure from HWE as a distance from the diagonal line in SO and hybrids (Figure S11C -E), while in BO the two estimators are almost the same, indicating BO are ideally under individual level of HWE (Figure S11A and S11B).

Their major concern in Manichaikul et al. 2010 was that the violation of HWE in the direction of too little homozygosity (due to reasons such as genotyping errors, recent admixture in a mixed population, or removing Mendelian errors in families) makes the estimator (Equation 9) over-estimate phi. To correct the effect of violated HWE, they used the smaller (the better) of the observed heterozygosity rates of a pair of samples (the number of heterozygous sites in an individual divided by the total number of non-missing markers for the pair of individuals) as an alternative to as expected heterozygosity for Equation (11).

In our case, the major concerns of violation of HWE are the extremely low genetic diversity of SO predicted from their current endangered status and the decreased homozygosity in hybrids due to the recent admixture. To mitigate these effects of violated HWE, we used the larger of the observed heterozygosity rates of a pair involving a SO sample as an alternative to expected heterozygosity in Equation (11) and used the smaller for a pair that doesn’t involve SO. For the pairs involving a SO and a hybrid samples, phi values are potentially inflated, since the too high heterozygosity of hybrids would be used as the larger heterozygosity in a pair. To examine this possible inflation of phi, we used the number of segregating sites and the number of zero IBS sites.

First, we searched for closely related samples within 2^nd^ degree relatives in the pairs of individuals within NSO (Figure S12A), CSO (Figure S12B), EBO (Figure S12C), and WBO (Figure S12D). No related individuals were detected from the kinship coefficient for NSO, CSO, and EBO. The absence of parent-offspring pairs in these populations was also confirmed the fact that all the IBS0 values of these populations are significantly larger than 0. For the pairs in WBO (Figure S12D), six pairs of parent-offspring or siblings were identified by phi. Among the six pairs, four showed almost 0 of IBS0 values (5.73 x 10^-6^ – 1.75 x 10^-5^), while two showed significantly larger values (2.76 x 10^-2^ and 4.02 x 10^-2^). We concluded that the former are parent-offspring pairs and the latter are siblings. The small phi values of the four pairs would correspond to genotyping errors. For the two pairs of siblings, we identified the pair of ZRHG114 and ZRHG123 (phi = 0.248) as full siblings and the pair of ZRHG126 and ZRHG127 (phi = 0.127) as close relatives, likely half-siblings, since the expected phi value for full siblings is 0.25 and that for half-siblings is 0.125 (Table S7). We parsimoniously removed four samples, ZRHG101, ZRHG123, ZRHG124 and ZRHG127 from our analyses of demography and genetic diversity not to leave related pairs in a population (Table S7B).

Next, we searched for related individuals in the pairs between BO and hybrids and pairs between SO and hybrids to supply detailed information about relatedness among the specimens in our sample set for future conservation studies. For the pairs between BO and hybrids, no close relatives were found (Figure S13A and S13B), while for the pairs between SO and hybrids, a cluster of pairs was detected as parent-offspring or siblings with phi (Figure S13C and S13D). Among them, four pairs of an NSO and a hybrid in the cluster show almost no zero IBS sites (Figure S13E), indicating they are parent-offspring pairs, while all pairs of a CSO and a hybrid showed a significantly larger proportion of zero IBS sites than 0 (Figure S13F). As we explained above, phi values between SO and hybrids are possibly inflated, so we examined the clusters with high phi value (>0.2) in details with the number of segregating sites (S) and the number of sites where no alleles are shared between two individuals in a pair (N_AAaa_). We found that among the 88 pairs between NSO and hybrid, all the 64 pairs involving an F1 hybrid clustered at IBS0 <0.1 (Figure S13C, S13E, Figure S14), reflecting their low genetic diversity. All the pairs except for the four parent-offspring pairs showed only the average level of S and N_AAaa_ (Figure S14), suggesting these 60 pairs are not related. It is also supported by the fact that backcrosses are not involved in these 60 pairs because non-parent-offspring related pairs should involve backcrosses, not F1 hybrids. We concluded that the high kinship coefficient values of these 60 pairs are inflated by the violation of Hardy-Weinberg equilibrium. Similary, among the 33 pairs between CSO and hybrid, all the 24 pairs involving an F1 hybrid clustered at IBS0 <0.1 (Figure S13D, S13F, Figure S14). We examined these pairs and showed none of them are related (Figure S14). In total, we identified 8 parent-offspring pairs involving 4 different parents and 8 offspring, one pair of full siblings, and one pair of closely related individuals, possibly half siblings (Table S7).

Additionally, we check if there are parent-offspring pairs between F1 and backcross in our samples using number of zero IBS sites. Generally, number of zero IBS sites is intermediate (~ 100s) between F1 individuals because they have one WBO allele and one EBO allele at tall the sites with fixed difference between WBO and EBO and because they cannot be parent-offspring pairs. All the pairs between F1 and a backcross in our sample showed large number of zero IBS sites though the number of segregating sites was similar with the pairs within F1 (Figure S15), revealing that they are not parents and offsprings.

**2. ADMIXTURE analysis**

We ran ADMIXTURE (Alexander et al. 2009) to provide another approach to see ancestry in hybrids in addition to our analysis using the fixed differences between SO and BO and between CSO and NSO (Fig 2D). We removed five samples which have closely related samples in our data set, which are ZRH615, ZRH625, ZRHG101, ZRHG123, ZRHG124, ZRHG127 (Table S7B) to avoid having relatedness dominant over ancestry and population structure. Using the remaining 45 samples, we thinned the data removing each SNP that has an r2 value of greater than 0.1 with any other SNP within a 50-SNP sliding window. Then we ran ADMIXTURE under a range of K-values (K=1-5).

As a result, the most likely number of genetic components was K=2 (Figure S8A), showing a clear separation between components from SO and BO (Figure S8B) consistent with the result using fixed alleles (Figure 2D). In the run with K=3, EBO and WBO were separated, while the structure within WBO and the structure within EBO became visible additionally with K= 4 and K=5, respectively. The partition between CSO and NSO could not be observed under the range of K-values (K=1-5). Because the relative number of sites which are involved in separations of groups affects the results in ADMIXTURE, it is likely to be the reason why it failed to detect the separation between CSO and NSO that the number of sites separating these two subspecies was too small compared with the number of sites involved in the separations of population structures in BO due to the decreased diversity in SO. When we ran ADMIXTURE using spotted owls only (11 individuals), separation between CSO and NSO was observed under K=2 with introgression of CSO component to NSO (Figure S8D), though the most likely number of genetic components was K=1 (Figure S8C).

**3. Tajima’s D for populations**

Tajima’s D values and the number of segregating sites for each population is shown in Table S10. For spotted owls, Tajima’s D were negative (-0.47 ±1.06 for SO and -0.63 ±1.03 for NSO), suggesting that they experienced population expansions sometime ago, and that their decline in population size is too recent (starting ~100 years ago) to be reflected to Tajima's D as a positive value. CSO showed a positive value of Tajima's D, but we must note that the number of samples of them is small (n = 3) and they showed the highest variance. For barred owls, it is believed that they have kept a sufficiently large population size so far, and recent shrinkage of population size is not known. Consistent with this, Tajima's D for the entire barred owl population and eastern barred owls were negative (-0.35±0.28 for BO and -0.52±0.25 for EBO). Western barred owls showed positive value (0.21±0.35), which is consistent with a founder event for WBO involving a small number of migrants from the eastern populations.

**4. mtDNA analyses**

The work of Barrowclough et al. 2011 has shown that there are two distinct mitochondrial haplotypes with different geographical distribution in barred owls, using 121 mitochondrial control-region sequences (518 bp) sampled form 18 populations distributed across the United States and Canada. They suggested two populations in the past, possibly two Pleistocene refugia, one was located on the Atlantic Coast and the other was in the south-central part of United States. The two ancient populations represented by these mitochondrial haplotypes have been spread and merged around the boundary areas probably after the last ice age. Barrowclough et al. 2011 also reported that both of the haplotypes were found on the west coast of the United States and in the boundary area, ranging from Minnesota, Michigan, Ohio to Florida. Although we don’t know how long the two populations were separated from each other, nor when they started to merge, the autosomal genetic diversity harbored by the two populations should have been rigorously mixed by recombination since the two populations encountered each other in the boundary area and spreading to the entire barred owl population with gradient proportions.

To confirm that our data contain the genetic components from both of the two ancient populations, we examined haplotypes of mtDNA in our data.

4-1. Extraction of mitochondrial variants

To obtain variants on mitochondrial DNA (mtDNA), we filtered the masked file separately from the nuclear variants using the GATK SelectVariants tool with the "--restrictAllelesTo BIALLELIC --excludeFiltered" options, and then extracted the variants mapped to the mitochondirial genome sequence in the reference file using vcftools (Danecek et al. 2011). We filtered the resulting file and eliminated the sites with the minimum quality of assigned genotype (GQ) smaller than 40. At each site, we took an allele with higher read depth between reference and alternative, and used the alleles with higher read depth than 20.

4-2. Phylogenetic analysis of the mitochondrial non-coding region

In previous works, the mitochondrial control region was used for studies of the phylogeography of spotted owls and barred owls (Haig, Mullins, Forsman, et al. 2004; Barrowclough et al. 2005; Barrowclough et al. 2011), but Hanna et al. (2017)(Hanna et al. 2017) has elucidated that mitochondria of spotted owls and barred owls have duplicated control regions. Because NGS short reads techniques don’t give the best quality data for duplicated or repetitive sequences, and variants on them are quite often mapped to wrong copies, we decided to remove the control regions and the genes between them (Figure S16). Instead, we used all the remaining non-control region of mtDNA (15kb) for the phylogenetic analysis with MEGA(Kumar et al. 2016). After removing all the sites with missing individual data, the remaining 327 variants on mtDNA for 51 male and female samples were used. Evolutionary histories were inferred using the Neighbor-Joining method (Saitou and Nei 1987), and the evolutionary distances were computed using the number of differences method (Nei and Kumar 2000) and are in the units of the number of base differences per sequence.

The resulting phylogenetic tree of the mitochondrial non-control region in our data showed clear clusters of SO and BO (Figure S9). In the barred owl cluster, two clades were formed and both of them involve EBO and WBO, supported by higher bootstrap values of 99 and 95. The nucleotide diversity and Hudson’s Fst between these two haplotypes on mtDNA were 0.0047 and 0.547 respectively. In the SO clade, two of the three CSO samples, UWBM62061 and ZRHG104, showed the deepest split from the rest, but the other CSO individual, ZRHG103, clustered together with NSO samples (Figure S9). Considering that ZRHG103 is from a hybrid zone between CSO and NSO, in Nevada County in California, it suggests that the individual carries an introgressed mtDNA from NSO, though we need more samples to examine it. Previous works based on mtDNA have reported the presence of CSO haplotypes in the range of NSO, and vice versa (Fleischer et al. 2004; Haig et al. 2004; Barrowclough et al. 2005), and the work on microsatellite loci showed that both long-distance dispersal and hybridization are occurring (Funk et al. 2008). Our result of this mtDNA analysis together with the ancestry analysis of hybrids supports hybridization between CSO and NSO.

4-3. Identification of mitochondrial lineages

To identify the haplotypes reported by Barrowclough et al. 2011 in our data, we identified five fixed differences between “Atlantic Coast” and “south-central” haplotypes using their 121 mitochondrial control-region sequences (518 bp) (JN097839 – JN098025) with MEGA software. Among the five SNPs, we found two (position 14886 and 14947) in our vcf file, while the other three were filtered out. We checked the genotypes of 51 samples at these two sites in our data, and found that at position 14886, T and A are segregating in our data, while G and A are segregating in the 121 sequences, suggesting mis-mapping of reads. At position 14947, T and C are segregating in both sets of data. We identified the two mitochondrial haplotypes in Barrowclough et al. 2011 on our non-control region sequences with position 14947 (Figure S9). We identified 24 and 14 sequences as linked sequences to the Atlantic Coast and the south-central haplotypes of the control region, respectively. Although this identification depends on a single variant, these two haplotypes corresponded perfectly to the two clusters on the phylogenetic tree (Figure S9). The geographic distribution of these haplotypes was quite similar to the one shown using the control region (Fig S17 and Figure 1 in Barrowclough et al. 2011). These results revealed that our samples include the known population structure observed on mtDNA, suggesting that our data also contains the autosomal genetic variety accumulated in both of the two ancient populations at least partially. The genetic varieties accumulated in the two distinct ancient populations found in an individual might be the cause of the complicated SMC++ patterns for barred owls.

**5. Parameters for population split time estimation**

Uncertainty of generation time and mutation rate leave some inaccuracy in our estimation of population split time. If the actual mutation rate for barred owls is twice as much as the used mutation rate (4.6 x 10^-9^ / bp per generation in flycatchers), an effective population size derived from the pi and the mutation rate would be 60000 and the estimated split time calculated with the generation time of 5 years would be 3500 years (TableS12). If we take generation time possibly varying from 2 to 10 years into account (see Generation time for analyses in Materials and Methods) as well, the estimated split time would be 1400-7000 years. If the mutation rate is half, the effective population size would be 240000 and the estimated split time calculated with the generation time of 5 years would be 14000 years. It can vary from 5600 to 28000 years depending on generation time. However, even when allowing for uncertainty in our generation time estimate and mutation rate, our results (1400-28000 years) appear to be at odds with the commonly assumed scenario of a very recent divergence (i.e., within the past 80-130 years) of WBO from the EBO population.

**Captions for Supplementary Figures**

Figure S1. Comparison of the distribution of the lengths of scaffolds and contigs between the assemblies.

The improved contiguity of our new assembly (StOcCau_2.0) was shown in comparison with the previous one (StOcCau_1.0).

Figure S2. Histogram of the mean read depth of scaffolds (>1Mb) in males and females.

We calculated the averaged read depth for each scaffold across variants and individuals for males and females.

Figure S3. Histogram of the proportion of missing data in scaffolds and contigs (<1Mb, >=100kb) in males and females.

The mean proportion of missing data was calculated across individuals for each scaffold or contig (<1Mb, >=100kb), separately for males and females.

Figure S4. Description of variants identified on autosomes and sex chromosomes.

The mean proportion of missing data for each scaffold/contig was plotted for males against that in females for autosomes (A), the Z (B) and the W chromosome (C). The number of genotypes was plotted against the length of the scaffold/contig for males (blue) and females (orange), and for autosomes (D), the Z (E) and the W chromosomes (F).

Figure S5. Sampling locations of genetically identified samples.

Sampling locations of the 51 individuals in our study. Spotted owls (SO, *Strix occidentalis*), barred owls (BO, *Strix varia*) and hybrids identified with PCA plots (Figure2). For locations with a high density of samples (e.g. Humboldt County and Siskiyou + Shasta County in California; Lane + Benton County in Oregon), the distribution of sampled individuals is visualized in pie charts. The size of circles and pie charts correspond to the number of samples. The range of barred owls was shown in green. The ranges for NSO and CSO are shown with red and orange lines respectively.

Figure S6. PCA plot for barred owls.

PCA plot for WBO and EBO samples was shown with sample names.

Figure S7. Nucleotide diversity between EBO and WBO

1. Pi between a EBO sample and WBO samples.

Nucleotide diversity was calculated between each EBO sample and 13 WBO samples.

1. Pi between a WBO sample and EBO samples.

Nucleotide diversity was calculated between each WBO sample and 12 EBO samples.

Figure S8. ADMIXTURE analysis

The 45 unrelated samples were used for A and B, while 11 SO samples were used for C and D.

1. Cross validation error with different K values.
2. ADMIXTURE plots under models with different number of ancestral populations. Individuals were sorted into Spotted owls (SO), eastern barred owls (EBO), western barred owls (WBO), F1 hybrids (F1) and backcrosse with WBO (BC).
3. Cross validation error with different K values for SO.
4. ADMIXTURE plots for SO under models with different number of ancestral populations. Individuals were sorted into northern spotted owls (NSO) and California spotted owls (CSO).

Figure S9. The phylogenetic tree on the non-control region of mtDNA

Variants on the non-control region (15kb) of all the 51 samples were involved. The sum of the branch length was 337.19. After eliminating all positions containing gaps and missing data, there were a total of 327 positions in the final dataset. The evolutionary history was inferred using the Neighbor-Joining method. The percentages of replicate trees in which the associated taxa clustered together in the bootstrap test (1000 replicates) are shown when it’s higher than 50%. Atlantic Coast and the south-central haplotypes were identified using a SNP in a control region.

Figure S10. Schematic picture of private alleles and population structure.

## Trees schematically show population structure in barred owls. Branches in blue and magenta represent EBO and WBO samples in a group (described in the main text), respectively, while dots are mutations occurred on the branches. The actual number of EBO in a group is 12 but only 4 is shown here to save space. (A) If WBO diverged very recently from a panmictic EBO population, or (B) if WBO is just a subset of EBO population, the number of private alleles specific to the WBO sample (2) should be roughly the same as the mean number of private alleles in EBO samples (5/4=1.25). (C) If WBO population has been genetically isolated from EBO for long time, a significant number of mutations, which have been accumulated in WBO population since the split from EBO, would show up as private alleles in a single WBO sample in a group (here, 5).

Figure S11. Comparison of two estimators of the kinship coefficient.

The two estimations of the kinship coefficient (phi) calculated with Equation(9) and Equation(11) from Manichaikul et al. 2010 were compared. The two estimations would be identical (on the diagonal line) if there is no violation of HWE. Two values were compared for pairs of samples within EBO (A), WBO (B), NSO (C), CSO (D) and hybrids (E).

Figure S12. Inference of related individuals within populations.

Phi values are plotted against the proportion of zero IBS (the portion of the sites where two individuals share no alleles identical by state) for each pair of samples within NSO (A), CSO (B), EBO (C) and WBO (D). Dashed lines are inference criteria of phi (from Table 1 in Manichaikul et al. 2010) for parent-offspring pairs and full siblings (PO + FS) and 2^nd^ degree relations (2D).

Figure S13. Inference of related individuals between populations.

Phi values are plotted against the proportion of zero IBS (the portion of the sites where two individuals share no alleles identical by state) for each pair of samples involving EBO and hybrids (A), WBO and hybrids (B), NSO and hybrids (C), and CSO and hybrids (D). The squared parts in (C) and (D) were enlarged in (E) and (F) respectively. Dashed lines are inference criteria of phi (from Table 1 in Manichaikul et al. 2010) for parent-offspring pairs and full siblings (PO + FS) and 2^nd^ degree relations (2D).

FigureS14. Sampling location, number of segregating sites and number of zero IBS sites for the pairs with high phi values.

Sampling location, number of segregating sites (S) and number of zero IBS sites (N_AAaa_) were shown for the pairs with high phi and low IBS0 values involving a hybrid and a SO sample. All the 64 pairs of samples in FigS13 (E) and all the 24 pairs in the FigS13 (F) are shown in green. Among them, four parent-offspring pairs detected with IBS0 values are shown in pink. All the individuals involved in the parent-offspring pairs are from Humboldt County in California (shown in orange). The remaining sympatric pairs showed only the average level of S and N_AAaa_.

Figure S15. Number of zero IBS sites and segregating sites

for the pairs within F1 and between F1 and a backcross.

Figure S16. The mean DP and the number of missing individual data for variants on mtDNA.

The mean read depth (blue) and the number of missing individual data (orange) were shown. The region containing the duplicated control regions, two tRNA genes and the ND6 gene (position 14879~) was removed from the phylogenetic analysis.

Figure S17. Geographic distribution of the mitochondrial haplotypes.

Distribution of the Atlantic Coast (blue, 24 individuals) and the south-central (magenta, 14 individuals) haplotypes in our data were shown to be compared with Figure 1 in Barrowclough et al. 2011. The size of circles and pie charts correspond to the number of samples.

**Captions for Supplementary Tables**

TableS1. Metrics of Assemblies.

Summary statistics for our new assembly (StrOccCau_2.0) in comparison with the previous assembly (StrOccCau_1.0). We removed contigs and scaffolds shorter than 1 kb from our assembly before calculating these statistics to make it comparable to StrOccCau_1.0. N50, L50, the percentage of missing data (Ns) and the total length of scaffolds and contigs are shown together with the number of scaffolds and contigs longer than 1kb or 1Mb.

TableS2. Detail description of the 82 scaffolds identified on autosomes.

The mean read depth (DP), the mean number of variants, and the mean portion of missing data across samples for each of 82 scaffolds identified on autosomes were shown with their standard deviations. The variants from the filtered set of SNPs, after removing individual variants with GQ smaller than 40, were used for this table (as used in the diversity analysis). The values for the total 51 samples, and for males and females were shown and no significant difference between sexes were there.

TableS3. Detail description of the 15 scaffolds identified on the Z chromosome.

The mean portion of missing data, the mean read depth (DP), and the mean number of variants across samples for each of the 15 scaffolds identified on the Z chromosome were shown with their standard deviations for males and females. The variants from the filtered set of SNPs, after removing individual variants with GQ smaller than 40, were used to make the table comparable with Table S2.

TableS4. Detail description of the 44 scaffolds/contigs identified on the W chromosome.

The mean portion of missing data, the mean read depth (DP), and the mean number of variants across samples for each of the 44 scaffolds and contigs identified on the W chromosome were shown with their standard deviations for males and females. The variants from the filtered set of SNPs, after removing individual variants with GQ smaller than 40, were used to make the table comparable with Table S2.

TableS5. Sample information.

Short sample IDs used in this study were shown with corresponding museum specimen IDs. All samples were primarily identified by sampling location, morphology and vocalization (“Primary identification”), then re-identified using the genetic data (“Genetic identification”). Genetically identified sex (M; Male, F; Female), sampling locations, and the mean and the standard deviation of read depth across variants were shown. The mean and the standard deviation of read depth were calculated for variants on scaffolds longer than 1Mb after the basic filtering. The averaged read depth across the sample means was 31.7 and the standard deviation was 6.51.

TableS6. Ancestral components of putative and genetically identified hybrid samples.

A. The portion of “spotted owl alleles” in hybrids at the apparent fixed differences between SO and BO was shown. Heterozygosity at these sites and inferred status of hybrids are shown.

B. The portion of “NSO allele” and “CSO allele” at the apparent fixed differences between NSO and CSO, where no polymorphism was observed in BO, was shown.

TableS7. List of closely related samples.

1. Detected pairs of related samples were shown with their phi, the portion of the sites where two individuals share no alleles identical by state (IBS0), and inference of their relationship.

B. Parsimonious list of related samples. The four samples removed from the diversity analyses and demography analyses were marked with asterisks.

TableS8. Nucleotide diversity within and between populations on autosomal variants. A set of variants after filtering out the individual variants with GQ smaller than 40 was used.

1. Pi within population.
2. Pi between populations.

Table S9. Weir and Cockerham’s F_ST_ for each pair of populations.

Weir and Cockerham‘s weighted F_ST_ for each pair of populations was calculated using autosomal variants.

Table S10. The number of segregating sites and Tajima’s D.

The number of samples and the number of segregating variants in two different sets of variants were shown for each population. The set retaining all the individual variants with GQ greater than 40 was used for the diversity analysis, and the set retaining only the variants with no missing data was used for all the other analyses. The four WBO individuals those have closely related individuals in the sample set were removed here. Tajima’s D, and the averaged number of variants across the windows used to calculate Tajima’s D were also shown.

Table S11. The number of private alleles.

The number of private alleles (singletons and private homozygotes) (NP) in groups are shown for before (A) and after (B) removing exons. NP in a WBO individual in each group (NP_WBO) and the mean NP for EBO individuals (Mean NP_EBO) are also shown.

TableS12. Various parameters to estimate split time between populations.

A range of generation time estimated by previous works (2-10 years, see Generation time for analyses in Materials and Methods) are used to calculate split time between populations together with different mutation rates. Note that effective population sizes change depending on mutation rates.

**Supplementary reference**

Alexander DH, Novembre J, Lange K. 2009. Fast model-based estimation of ancestry in unrelated individuals. Genome Res. 19:1655–1664.

Barrowclough GF, Groth JG, Mertz LA, Gutiérrez RJ. 2005. Genetic structure, introgression, and a narrow hybrid zone between northern and California spotted owls (Strix occidentalis). Mol. Ecol. 14:1109–1120.

Barrowclough GF, Groth JG, Odom KJ, Lai JE. 2011. Phylogeography of the Barred Owl ( Strix varia ): Species limits, multiple refugia, and range expansion . Auk 128:696–706.

Danecek P, Auton A, Abecasis G, Albers CA, Banks E, DePristo MA, Handsaker RE, Lunter G, Marth GT, Sherry ST, et al. 2011. The variant call format and VCFtools. Bioinformatics 27:2156–2158.

Fleischer R, Dumbacher J, Moritz C, Monahan W. 2004. Assessment of the Subspecies and Genetics. In: SCIENTIFIC EVALUATION OF THE STATUS OF THE NORTHERN SPOTTED OWL. Portland, Oregon: Sustainable Ecosystems Institute.

Funk WC, Forsman ED, Mullins TD, Haig SM. 2008. Introgression and dispersal among spotted owl (Strix occidentalis) subspecies. Evol. Appl. 1:161–171.

Haig SM, Mullins TD, Forsman ED. 2004. Subspecific relationships and genetic structure in the spotted owl. Conserv. Genet. 5:683–705.

Haig SM, Mullins TD, Forsman ED, Trail PW, Wennerberg L. 2004. Genetic identification of spotted owls, barred owls, and their hybrids: Legal implications of hybrid identity. Conserv. Biol. 18:1347–1357.

Hanna ZR, Henderson JB, Sellas AB, Fuchs J, Bowie RCK, Dumbacher JP. 2017. Complete mitochondrial genome sequences of the northern spotted owl (Strix occidentalis caurina) and the barred owl (Strix varia; Aves: Strigiformes: Strigidae) confirm the presence of a duplicated control region. PeerJ 2017.

Kumar S, Stecher G, Tamura K. 2016. MEGA7: Molecular Evolutionary Genetics Analysis Version 7.0 for Bigger Datasets. Mol. Biol. Evol. 33:1870–1874.

Manichaikul A, Mychaleckyj JC, Rich SS, Daly K, Sale M, Chen WM. 2010. Robust relationship inference in genome-wide association studies. Bioinformatics 26:2867–2873.

Nei M, Kumar S. 2000. Molecular evolution and phylogenetics. Oxford: Oxford University Press

Saitou N, Nei M. 1987. The neighbor-joining method: a new method for reconstructing phylogenetic trees. Mol. Biol. Evol. 4:406–425.

| Assembly | N50 (kb) | L50 | Ns (%) | Total length of scf/ctg  >1kb (Mb) | Number of scf/ctg  > 1kb | Total length  of scf/ctg >1Mb (Mb) | Number  of scf/ctg  > 1Mb |
| --- | --- | --- | --- | --- | --- | --- | --- |
| StrOccCau_1.0 | 3,983.0 | 92 | 1.10 | 1,255.5 | 8108 | 1,075.5 | 303 |
| StrOccCau_2.0 | 20,549.8 | 16 | 1.91 | 1,254.4 | 11568 | 1,173.7 | 97 |

**Supplementary tables**

TableS1. Metrics of Assemblies.

Table S6. Ancestral components of putative and genetically identified hybrid samples.

A. Portion of SO ancestry in hybrids

| ID | Portion of SO ancestry | Heterozygosity | State |
| --- | --- | --- | --- |
| 1957-00137 | 0.500 | 1.000 | F1 |
| ZRH962 | 0.500 | 1.000 | F1 |
| 1957-00240 | 0.500 | 1.000 | F1 |
| ZRH610 | 0.500 | 1.000 | F1 |
| ZRH600 | 0.500 | 0.999 | F1 |
| 1957-00243 | 0.500 | 0.999 | F1 |
| LCW1363 | 0.500 | 0.999 | F1 |
| LCW1383 | 0.499 | 0.997 | F1 |
| TLW519 | 0.322 | 0.644 | F1 x BO |
| ZRH607 | 0.276 | 0.551 | F1 x BO |
| TLW528 | 0.241 | 0.481 | F1 x BO |
| AFRD90 | 0.000 | 0.000 | BO |
| TLW532 | 0.000 | 0.000 | BO |
| TLW521 | 0.000 | 0.000 | BO |
| CYWC009 | 0.000 | 0.000 | BO |

Table S6. Ancestral components of putative and genetically identified hybrid samples.

B. Portion of NOS/CSO ancestries in hybrids

| State | ID | Portion of NSO ancestry | Portion of CSO ancestry | Total |
| --- | --- | --- | --- | --- |
| F1 | 1957-00137 | 0.500 | 0.000 | 0.500 |
|  | ZRH962 | 0.500 | 0.000 | 0.500 |
|  | 1957-00240 | 0.500 | 0.000 | 0.500 |
|  | ZRH610 | 0.500 | 0.000 | 0.500 |
|  | ZRH600 | 0.479 | 0.021 | 0.500 |
|  | 1957-00243 | 0.466 | 0.034 | 0.500 |
|  | LCW1363 | 0.256 | 0.244 | 0.500 |
|  | LCW1383 | 0.226 | 0.274 | 0.500 |
| F1 x BO | TLW519 | 0.245 | 0.078 | 0.322 |
|  | ZRH607 | 0.209 | 0.066 | 0.276 |
|  | TLW528 | 0.136 | 0.105 | 0.241 |
| BO | AFRD90 | 0.000 | 0.000 | 0.000 |
|  | TLW532 | 0.000 | 0.000 | 0.000 |
|  | TLW521 | 0.000 | 0.000 | 0.000 |
|  | CYWC009 | 0.000 | 0.000 | 0.000 |

Table S7. List of closely related samples.

1. Full list of closely related pairs.

|  | Individual 1 | Individual 2 | Phi | IBS0 | Inference |
| --- | --- | --- | --- | --- | --- |
| Within BO | ZRHG101 | ZRHG119 | 0.248 | 5.73E-06 | Parent - offspring |
|  | ZRHG101 | ZRHG126 | 0.246 | 8.10E-06 | Parent - offspring |
|  | ZRHG114 | ZRHG123 | 0.213 | 4.02E-02 | Full siblings |
|  | ZRHG114 | ZRHG124 | 0.252 | 1.75E-05 | Parent - offspring |
|  | ZRHG123 | ZRHG124 | 0.260 | 1.37E-05 | Parent - offspring |
|  | ZRHG126 | ZRHG127 | 0.179 | 2.76E-02 | Half siblings |
| Hybrid - SO | 1957-00137 | ZRH615 | 0.250 | 6.48E-06 | Parent - offspring |
|  | 1957-00240 | ZRH625 | 0.250 | 5.45E-06 | Parent - offspring |
|  | ZRH610 | ZRH615 | 0.250 | 1.22E-05 | Parent - offspring |
|  | ZRH962 | ZRH615 | 0.250 | 7.25E-06 | Parent - offspring |

B. Parsimonious list of related samples.

| ID | Population | Related individuals |
| --- | --- | --- |
| ZRH615 | NSO | Father of ZRH610 (F1), 1957-00137 (F1) and ZRH962 (F1) |
| ZRH625 | NSO | Father of 1957-00240 (F1) |
| ZRHG101* | WBO | Offspring of ZRHG119 (WBO) and ZRHG126 (WBO) |
| ZRHG124* | WBO | Parent or offspring of ZRHG114 (WBO) and ZRHG123 (WBO) |
| ZRHG123* | WBO | Full sibling of ZRHG114 (WBO) |
| ZRHG127* | WBO | Closely related samples (potentially half-siblings) of ZRHG126 (WBO) |

TableS8. Nucleotide diversity within and between populations on autosomal variants.

A. Pi within population

| Population | Pi |
| --- | --- |
| SO | 1.41.E-04 |
| NSO | 1.14.E-04 |
| CSO | 1.48.E-04 |
| BO | 2.32.E-03 |
| WBO | 2.15.E-03 |
| EBO | 2.37.E-03 |

1. Pi between populations

| Pop1 | Pop2 | PI |
| --- | --- | --- |
| SO | BO | 6.65.E-03 |
| SO | WBO | 6.64.E-03 |
| SO | EBO | 6.64.E-03 |
| NSO | CSO | 1.69.E-04 |
| NSO | BO | 6.65.E-03 |
| NSO | WBO | 6.64.E-03 |
| NSO | EBO | 6.64.E-03 |
| CSO | BO | 6.63.E-03 |
| CSO | WBO | 6.63.E-03 |
| CSO | EBO | 6.63.E-03 |
| EBO | WBO | 2.37.E-03 |

TableS9. Weir and Cockerham’s F_ST_ for each pair of populations.

| Pop1 | Pop2 | Weighted F_ST_ |
| --- | --- | --- |
| SO | BO | 0.765 |
| SO | WBO | 0.818 |
| SO | EBO | 0.806 |
| NSO | CSO | 0.253 |
| NSO | BO | 0.750 |
| NSO | WBO | 0.799 |
| NSO | EBO | 0.785 |
| CSO | BO | 0.713 |
| CSO | WBO | 0.748 |
| CSO | EBO | 0.727 |
| EBO | WBO | 0.050 |

TableS10. The number of segregating sites and Tajima’s D.

| Population | Number of samples | Number of segregating variatns (GQ>=40) | Number of segregating variants (GQ>=40) without missing data | Tajima'sD (Std) | Mean number of variants in a100kb window (Std) |
| --- | --- | --- | --- | --- | --- |
| SO | 11 | 539,226 | 456,255 | -0.470 (1.055) | 41.8 (24.6) |
| CSO | 3 | 312,929 | 300,698 | 0.146 (1.094) | 27.5 (24.6) |
| NSO | 8 | 409,928 | 360,001 | -0.634 (1.028) | 32.9 (23.6) |
| BO | 25 | 11,360,737 | 8,572,962 | -0.351 (0.284) | 784.6 (278.6) |
| WBO | 13 | 7,654,945 | 6,474,001 | 0.211 (0.349) | 592.5 (224.7) |
| EBO | 12 | 9,810,520 | 8,550,852 | -0.516 (0.254) | 782.5 (285.1) |

TableS12. Various parameters to estimate split time between populations.

| Generation time (y) | Ne | | Mutation rate | Estimated split time (y) | |
| --- | --- | --- | --- | --- | --- |
| 2 | 60000 |  | 9.2 x 10^-9^ | 1400 |  |
| 2 | 120000 |  | 4.6 x 10^-9^ | 2800 |  |
| 2 | 240000 |  | 2.3 x 10^-9^ | 5600 |  |
| 2 | 300000 |  | 1.84 x 10^-9^ | 7000 |  |
| 5 | 60000 |  | 9.2 x 10^-9^ | 3500 |  |
| 5 | 120000 |  | 4.6 x 10^-9^ | 7000 |  |
| 5 | 240000 |  | 2.3 x 10^-9^ | 14000 |  |
| 5 | 300000 |  | 1.84 x 10^-9^ | 17500 |  |
| 10 | 60000 |  | 9.2 x 10^-9^ | 7000 |  |
| 10 | 120000 |  | 4.6 x 10^-9^ | 14000 |  |
| 10 | 240000 |  | 2.3 x 10^-9^ | 28000 |  |
| 10 | 300000 |  | 1.84 x 10^-9^ | 35000 |  |
